# Delayed Response and Biosonar Perception Explain Movement Coordination in Trawling Bats

**DOI:** 10.1371/journal.pcbi.1004089

**Published:** 2015-03-26

**Authors:** Luca Giuggioli, Thomas J. McKetterick, Marc Holderied

**Affiliations:** 1 Bristol Centre for Complexity Sciences, University of Bristol, Bristol, United Kingdom; 2 School of Biological Sciences, University of Bristol, Bristol, United Kingdom; 3 Department of Engineering Mathematics, University of Bristol, Bristol, United Kingdom; Northeastern University, United States of America

## Abstract

Animal coordinated movement interactions are commonly explained by assuming unspecified social forces of attraction, repulsion and alignment with parameters drawn from observed movement data. Here we propose and test a biologically realistic and quantifiable biosonar movement interaction mechanism for echolocating bats based on spatial perceptual bias, i.e. actual sound field, a reaction delay, and observed motor constraints in speed and acceleration. We found that foraging pairs of bats flying over a water surface swapped leader-follower roles and performed chases or coordinated manoeuvres by copying the heading a nearby individual has had up to 500 ms earlier. Our proposed mechanism based on the interplay between sensory-motor constraints and delayed alignment was able to recreate the observed spatial actor-reactor patterns. Remarkably, when we varied model parameters (response delay, hearing threshold and echolocation directionality) beyond those observed in nature, the spatio-temporal interaction patterns created by the model only recreated the observed interactions, i.e. chases, and best matched the observed spatial patterns for just those response delays, hearing thresholds and echolocation directionalities found to be used by bats. This supports the validity of our sensory ecology approach of movement coordination, where interacting bats localise each other by active echolocation rather than eavesdropping.

## Author Summary

Collective movements of flocking birds or shoaling fish are amongst the most fascinating natural phenomena, and everyone has experienced the challenges of walking through a moving crowd. What information individuals use for movement coordination is, however, very difficult to know, except for echolocating bats. These flying mammals perceive their surroundings by emitting loud and high-pitched biosonar calls and listening for the returning echoes. Because bat biosonar imaging is much sparser in information than vision, we can accurately measure the biosonar calls of interacting bats with a group of microphones and then calculate what each of the individuals perceived. When observing pairs of Daubenton’s bats foraging low over water for stranded insects, we found they have intriguing ‘traffic rules’—they chase each other, perform tandem turns and even slow down to avoid collision. When we then modelled their biosonar view of the surroundings during these interactions we discovered that one simple trick suffices to create all their interactive behaviours: once another individual is close enough for your biosonar to pick up its echo, copy this individual’s flight direction within 4–5 of your own wingbeats.

## Introduction

Group movement patterns are dependent on perceptual inputs, governed by cognitive mechanisms, and constrained by the motor abilities of the interacting agents [1–4]. The intrinsic sequential occurrence of these three stages gives rise to delayed movement responses [5]. Current collective movement interpretations acknowledge such delays [6–9], but to explain observed patterns the existence of generic, albeit plausible, interaction rules are postulated. Since individual decisions depend on the information about the actions of other conspecifics [10–14] and movement response are shaped by an animal’s sensory-motor abilities, it is however biologically most realistic to explain interaction exclusively from perception biases and delayed responses without inferring social forces from the observed movements.

Even though various models are capable of capturing many of the emerging features of coordinated movement patterns, the individual behavioural rules are heuristics of the underlying mechanisms. There is awareness that more biologically oriented experimental analysis and modelling is necessary [15]. Recent experimental studies on golden shiners (*Notemigonus crysoleucas*) [16] have indicated that speed regulation along the direction of travel appears a more dominant component of the interaction compared to alignment, whereas studies in mosquitofish reported the lack of any justifiable alignment rule [17]. From three-dimensional stereographic images of starling flocks [18], evidence of a topological rather than a metric interaction rule was brought to the fore, i.e. individuals interacted with an average of six or seven neighbours rather than every conspecific within a certain distance. Other network topologies have also been proposed recently based on animal communication across a ‘Voronoi shell’ of nearest-neighbours [19] or the network of visible neighbours estimated from a ray-casting algorithm around a fish eye [20]. Other investigations have also questioned the assumptions about how individuals perceive and integrate information from their neighbours. Experiments on saithe (*Pollachius virens*) pointed to the importance of the use of lateral line compared to vision on school structure and dynamics [21].

All these recent efforts point to the importance of sensory inputs that shape an animal’s movement decisions. Animal species that use an active sensory modality, such as echolocation, offer an opportunity to tap directly into the source of this sensory information. Active-sensing, compared to passive, requires the emission of a signal whose spatial propagation is governed by quantifiable physical laws [22]. Therefore, once the call intensity and echolocation directionality of an echolocating species are known, it is possible to infer with sufficient accuracy over what spatial range individuals can detect and track each other, either by active sensing or, over greater distances, by eavesdropping on the others’ signals [23–25].

Here we introduce and test the biosonar movement interaction (BSMI) hypothesis, whereby bats interact with conspecifics, to perform coordinated flight, based on the information collected by their biosonar and not by eavesdropping. While foraging, bats send out sonar search pulses several times per second. Whenever a sound pulse hits an object an echo is generated, which then travels back to the bat who interprets the echo for target detection, recognition and localisation. Because real objects do not reflect all the impinging sound back to the echolocator (target strength < 1) and because the sound has to travel the bat-object distance twice incurring double propagation losses, the biosonar survey range is limited to several meters at best (see e.g. [24, 26, 27]). The spatial profile of biosonar is therefore determined by the amplitude of the biosonar pulses and by the directionality of sound emission as well as the directionality of bat hearing, where the biosonar detection range is greatest on axis and drops off towards either side. The core of the BSMI hypothesis is thus that the directional spatial profiles of the individuals’ biosonar fields determine the animals’ mutual positions and headings during movement interactions, with the information obtained via eavesdropping having a minor role in movement coordination. Note that by eavesdropping we mean locating conspecifics by listening to their echolocation calls [23] and not locating oneself by trailing a conspecific and extracting information from the echoes resulting from their calls [28].

Sound field emission profiles of echolocating bats have been documented in various species [29] including Daubenton’s bat, *Myotis daubentonii*, our study animal. This widespread medium-sized species [30] has been selected for two reasons. On one hand, the animal’s cognitive abilities allow for rich behavioural patterns. On the other hand, its habit to forage low over still water surfaces to glean arthropod prey [31] reduces the spatio-temporal complexity of the interactions to just a two-dimensional plane.

To test our BSMI hypothesis we collected movement trajectories of Daubenton’s bat pairs foraging freely in the wild. Based on these observations, we proceeded in three steps: (**i**), (**ii**), and (**iii**). First, we built a behavioural classifier (**i**) based solely on the paired movement paths, which segments these movement trajectories into different behaviours, and determines actor-reactor roles by extracting response delays. We then tested the BSMI hypothesis by comparing the relative positions of the interacting bats with the calculated biosonar perception field (**ii**). Finally, we constructed and tested a BSMI model of interacting bats (**iii**) whereby the mechanisms of interaction are based exclusively on aerodynamic constraints. i.e. actual speed and acceleration, and sensory inputs, i.e. the known spatial extent of the sonar perception field. The quality of the BSMI model is judged against its ability to reproduce the observed spatio-temporal interaction dynamics of the bats.

## Results

We recorded flight interactions of foraging Daubenton’s bats flying in the South-West corner of Barrow Tanks reservoir number three near Barrow Gurney in England. The experimental set-up allowed for a 20 ms temporal resolution of flight tracking (see Methods section). The recordings were taken over 10 evenings in the summer of 2009: June 15^th^, 22^nd^, 29^th^; July 1^st^, 7^th^, 8^th^, 9^th^, 15^th^; and August 19^th^ and 25^th^. Overall we collected nearly 70,000 individual data points (fixes) including 70 interactions as displayed in Table 1.

**Table 1:** Summary of the entire experimental dataset.

	Individual Flight	Paired Flight
Data points	60202	9134
Trajectories	405	70
Coordinated Flight (% of data)	–	22.35
Coordinated Flight (contributing trajectories)	–	28
Chase Flight (% of data)	–	4.11
Chase Flight (contributing trajectories)	–	9
Mean Time Flight (s)	2.95 ± 2.16	1.28 ± 1.19
Mean Flight Distance (m)	13.20 ± 9.60	12.59 ± 12.33
Mean Flight Speed (ms^−1^)	4.46 ± 1.51	4.89 ± 2.14

Breakdown of the original spatio-temporal data into the individual and paired flight trajectories together with mean and standard deviation values of the temporal and spatial length as well as speed of all flights.

To identify interacting flight behaviour we used two mathematical tools: the *time-dependent delayed correlation* (TDDC) and the *time-dependent delayed separation* (TDDS). For a given point in time, the TDDC compares the heading of one of the bats with the heading of the other at all other times, thus allowing us to establish whether one bat was copying the direction of the other (coordinated flight). Similarly the TDDS compares the position of one of the bats at one time with the position of the other bat at all other times, thus allowing us to specify if one bat was copying the position of the other (chase flight). The remaining data, when no indication of interaction emerged, were deemed unclassified.

**Fig 1:**
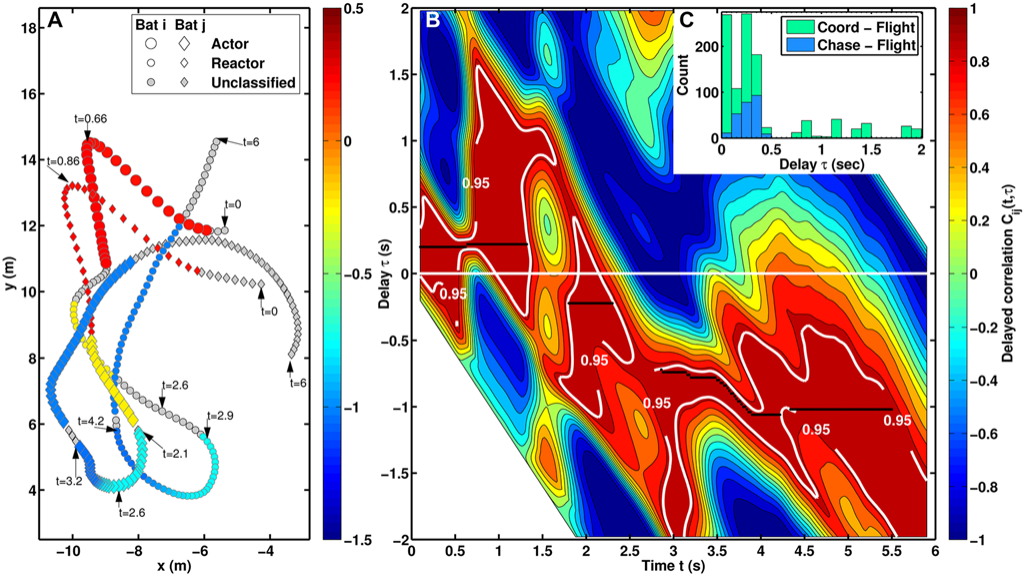
Sample spatio-temporal paired trajectory with the corresponding TDDC function, and observed delay values. (A) Example of a paired flight trajectory subsampled for clarity with every other data point from the original 20 ms resolution. Non-grey symbols show interacting flight segments classified as either chase or coordinated flight extracted (see Methods) from the analysis of the TDDC plot, shown in panel (B), and the TDDS plot (not shown). For a given time and delay the TDDC plot shows the correlation between the headings of the two bats (1 is perfect alignment, 0 occurs when the relative headings is ±90°, -1 represents opposite alignments). Regions of the TTDC plot inside the area demarcated by the 0.95 correlation threshold correspond to relative heading values smaller than 18.2° in magnitude. The colour-coding of the movement paths is obtained from the coordinates (*t*, *τ*) of the disjoint black lines (time-ordered procedure) in panel (b), which assign delay response values between the bats at each instant in time. When no black line is present in the TDDC plot, the behaviour is deemed unclassified and the corresponding movement path in panel (a) is drawn grey. Panel (C) displays the observed delay values of coordinated and chase flights from *all* recorded trajectories through the analysis of the corresponding TDDS and TDDC plots.

In Fig. 1A we show a sample trajectory (shown also in S1 Video), whose reaction delays have been extracted from the time-dependent delay directional correlation (TDDC) plot displayed in Fig. 1B—the corresponding time-dependent delay separation (TDDS) plot is not shown in this example as it reveals absence of chase behaviour. The presence of a black dot with coordinates (*t*, *τ*) in the *C*_*i*,*j*_(*t*, *τ*) plot shows that bat *j* has copied the heading of bat *i* with a delay *τ* at time *t*. A straight black line segment indicates that the bats have maintained a constant reaction delay over a certain period of time, whereas an inclined line, e.g. in the intervals 3.14 s < *t* < 3.20 s and 3.50 s < *t* < 3.88 s, illustrates an interaction with changing delay. If the extracted delay turns negative, bat *i* now copies the heading of bat *j* and actor-reactor roles have swapped. In this sample trajectory, this swap has occurred in two stages. At *t* = 1.4 s bat *i* first terminates the nearly parallel flight it was maintaining with bat *j*, and subsequently, at *t* = 1.8 s, tries to align its heading with that of bat *j* (see caption of Fig. 1 for more details).

We analysed those flights which had more than one bat in the field of view of the camera at one time. Our analysis allowed us to classify each recorded data point from all seventy paired trajectories as non-interacting (74%) and interacting behaviour (26%), with the latter further subdivided into chases (4%) and coordinated flights (22%). Non-interacting is the definition given to flight segments where there is low correlation between the headings of the individuals. Coordinated flight is defined as a flight segment with high correlation between the headings of the individuals, and chase flight is a flight segment where one individual occupies the previous positions of another. Note that chase flights are a subset of coordinated flights. As shown in Fig. 1C chase flights and most coordinated flights were characterized by similar response delay values of up to 500 ms, which corresponds to 5–7 biosonar calls, i.e. information updates, at the known average call interval of 70–100 ms [32]. One marked distinction between chases and coordinated manoeuvres was in their leader-follower dynamics, synonym here of actor-reactor. During chase flights, the individual ahead was clearly the leader [33], as the follower was retracing the movement path of the individual in front. This leader-follower hierarchy was not equally prominent when bats were performing coordinated flights, for example the reactor was not necessarily behind the actor (see e.g. S1 Fig. panel (f)).

In summary to realise step (**i**) in the validation of our BSMI hypothesis, we reconstructed movement trajectories from videogrammetry data, analysed the corresponding TDDC and TDDS plots, and extracted the associated delay values. This allowed us to distinguish movement paths among non-interacting flights, chases and coordinated flights. For the last two categories, the sign of the extracted delay values provided the sought actor-reactor roles as a function of time for each interacting paired trajectory (see S1 Fig. panels (a)-(f) for a graphical representation of the 6 steps of this procedure).

**Fig 2:**
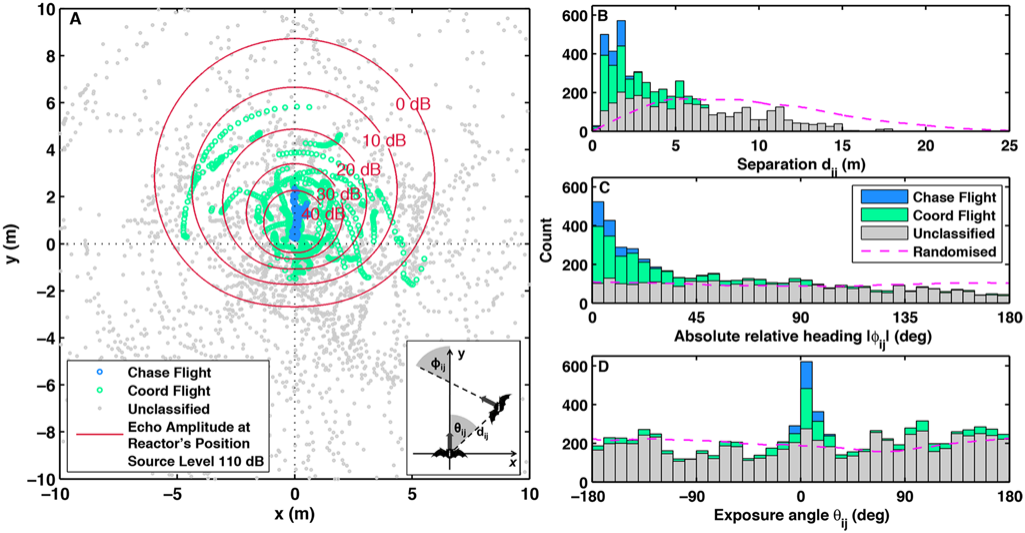
Bats’ relative locations with corresponding sound fields and summary statistics of all co-flying bats. Panel (A): relative positions of co-flying bats with reactor’s sound fields. Symbols indicate locations of one individual relative to the position (centred) and heading (upwards) of the other: blue for chases, green for coordinated flights, and grey for unclassified behaviour. In the latter case, the individual at the centre is picked at random for each pair. Reactor is in the centre with upward heading with symbols showing actor’s positions and with red lines representing isocontours of the reactor’s calculated echolocation field. Parameters: cosine directionality with front-rear difference of 36 dB accounting for both emission and hearing directionality (see Equation (11) below). Source level is 110 dB [29] and absorption is 1.28 dB m^−1^[22]. Inset indicates separation distances *d*_*ij*_, relative headings *ϕ*_*ij*_, i.e. the angular difference between the two velocity vectors, and exposure angles *θ*_*ij*_, i.e. the angular position of the actor with respect to the heading of the reactor. Panel (B), (C), and (D) display histograms of separation distances, absolute relative headings and exposure angles of co-flying bats obtained from panel (A).

To quantify alignment dynamics of the interacting bats, in Fig. 2 we plot bat separation distance, relative heading and relative position [19]. Chases were characterised by low separation distances (Fig. 2B), high alignment (Fig. 2C) and narrow frontal exposure angles (Fig. 2D), while coordinated flights occurred over larger separations, wider range of relative headings and only a moderate preference for frontal exposure angles (see S2 Fig. for the difference in the TDDS and TDDC plots between a coordinated flight and a chase). Unclassified data showed no preference in relative headings and exposure angles, yet comparison with randomised pairing of trajectories (dashed lines) revealed that unclassified data points were overrepresented at separations below 3.5 m and underrepresented above approximately 7 m. This indicated that even non-interacting bats tended to fly closer than random.

With the identified actor-reactor roles, it became possible to superimpose the bat’s echolocation field and test whether a plausible explanation for the observed interacting flights is the existence of an acoustic response threshold—step (**ii**) in our BSMI hypothesis. A match between the actual spatial patterns of interactions with these superimposed sound fields would indicate a potential functional link. In Fig. 2A, where we plotted the actor’s position relative to the heading and location of the reactor, we added isocontours of the calculated biosonar field, i.e. the amplitude of the echo reflected from the actor’s body returning to the reactor. We noticed that 99% of observations were above 0 dB and 97% above 10 dB, i.e. almost no reaction occurred when the actor’s echo was below the likely range for the reactor’s hearing threshold of 0–10 dB [34]. In short, there was no reaction when the actor was outside the reactor’s echolocation field.

**Fig 3:**
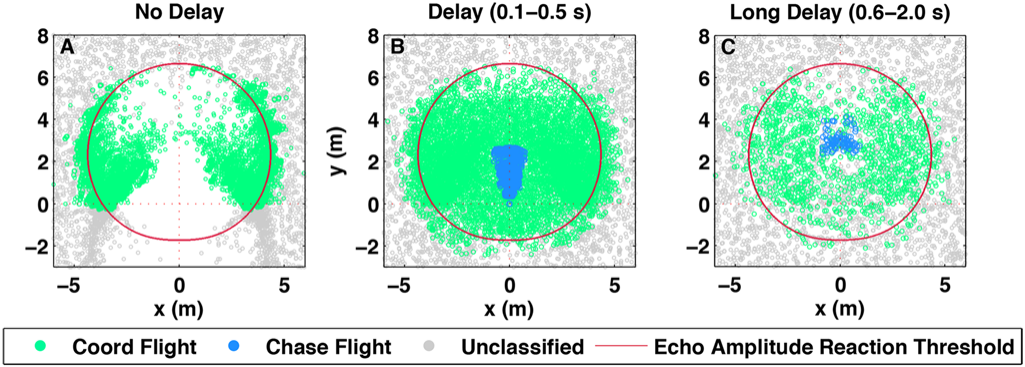
Model-generated spatio-temporal trajectories. BSMI model output of the actor’s locations relative to the reactor, according to the TDDC and TDDS analysis, for different delayed response scenarios, each with echo amplitude reaction threshold at 10 dB. The blue and green points represent chase and coordinated flight respectively. The grey points represent non-interacting flights for which, having neither actor nor reactor, one of the pair was randomly placed at the center. (A) Spatial patterns when alignment response is immediate; when delay values are multiple of 100 ms and up to 500 ms; and (C) with reaction delay values multiple of 100 ms and between 600 ms and 2 s. For clarity only around three thousand randomly selected grey points have been plotted in each panel. Only short delay values, panel (B), reproduce the observed spatial relationship in Fig. 2 where actors are aligned within the sound field contour and chasees occupy the forward position directly in front of the chaser. Panels (A) and (C) corresponding to the model with instant and long delays respectively, fail to produce the same populating of the sound field and have chases absent or present at greater separations than recorded in the field, respectively.

**Fig 4:**
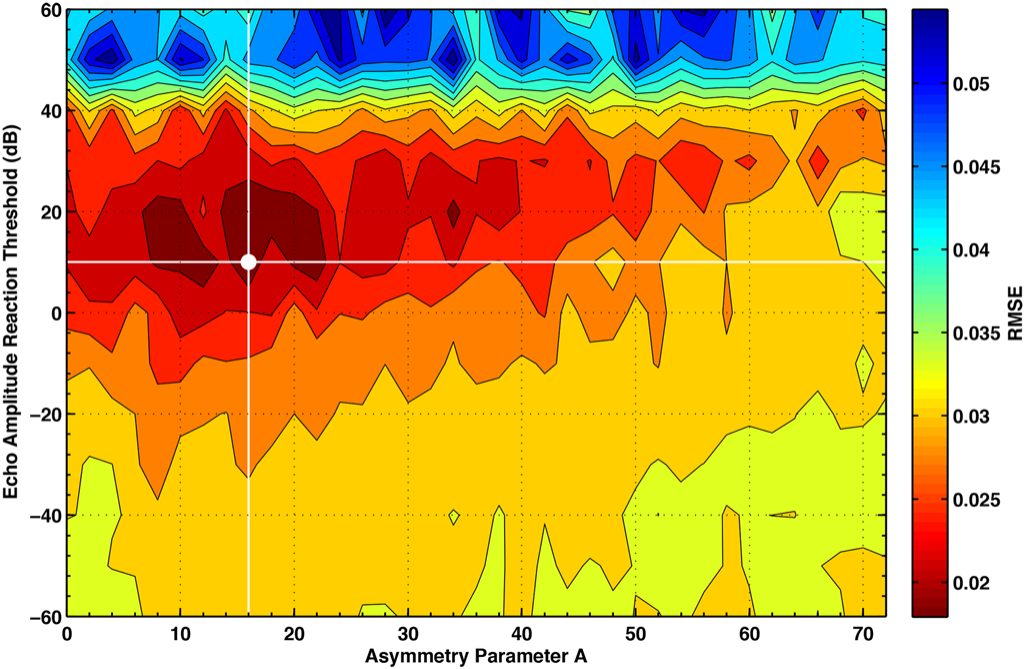
Fit of the model output to the observed interacting flights. Contour level plot representing the fit of the coordinated flight movement patterns with those obtained from the model. The fitting procedure consists of comparing the relative position plot of coordinated flights from Fig. 2A and from the model with 1.5 × 1.5 m^2^ resolution. For each grid square (*i*, *j*) the normalised probability density, 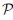
(*i*, *j*) is calculated and the lowest mean square error obtained via the expression 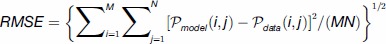. The best fit is marked by the white point and corresponds to the model interaction field with parameters *A* = 16 and *B* = 10 dB.

Step (**iii**) in our BSMI hypothesis consists of investigating whether alignment dynamics with delay in combination with the perceptual bias created by the shape of the echolocation field suffice to produce the observed behavioural interactions. For that purpose we constructed a mathematical movement model (BSMI model) of echolocating bat pairs capable of generating interacting trajectories that mimic the patterns of chases and coordinated flights observed in the field data (see Methods). From the spatio-temporal patterns emerging in the BSMI model, we generated relative position plots in Fig. 3 for different delay response *τ*, directionalities of the echolocation field—asymmetry parameter *A* in Equation (11) below—and hearing threshold—parameter *B* in Equation (12) below. By varying these parameters in the model we produced actor-reactor and chaser-chasee spatial relationships that can be compared to the observations in Fig. 2A. We found that the best fit was achieved for response delays between 100 ms and 500 ms, and with *B* = 10 ± 5 dB and *A* = 16 ± 1 (root mean standard error RMS = 0.0179), see Fig. 4. We used this latter value to calculate sound fields displayed in Figs. 2A and 3.

## Discussion

Movement analysis has a long tradition in animal ecology [35, 36], however, the segmentation and classification of trajectories of co-moving individuals is an area of research still in its infancy [37]. Here we have shown that a use of the velocity and separation delayed correlation maps, respectively the TDDC and TDDS functions, allowed us to classify interaction patterns in the movement trajectories of coflying bat pairs. Considering only those reactions that are strictly ordered in time, it is possible to reconstruct the delay with which one bat copies the heading of the other bat as a function of time.

With our automatic classifier we have found that bats foraging in the field move in coordination five times more often than chasing the bat in front. Although a chaser has a spatial advantage when either the leader fails to capture a prey item (2nd attacker advantage) or when a patch of resources is found (shared exploitation), a chase might also become an aggressive behaviour if the chaser directs unpleasant or even harmful loud signals at the chasee. On the other hand, coordinated flight could improve search efficiency because both individuals hear when and where the other has searched for and found prey, which can allow each member of the pair to survey larger areas of the water surface per unit of time through eavesdropping.

Empirical evidence strongly suggests that active echolocation was used by the reacting individual for alignment during interactions: 99% of all interactions were within the echolocation field and the entire echolocation field down to echo amplitudes of 0–10 dB was used (Fig. 2A). The possibility that eavesdropping per se might have produced the observed patterns was tenuous. We reached that conclusion by drawing the reactor’s position relative to the actor (see S3 Fig.). In that case no match of spatial interaction patterns with the received sound appeared obvious as interactions did not follow any sound exposure isocontour and most interactions happened in a direction opposite to the main lobe of emission. However, the fact that unclassified, i.e. non-interacting, bats flew at closer than random distances (Fig. 2B), often outside the maximum calculated echolocation range but within eavesdropping range (S3 Fig.), suggests the idea that it is beneficial for a bat to remain in the eavesdropping region of others, thereby possibly profiting from overhearing the other’s search success. Eavesdropping might also account for the low number of observations above -∣135∣° relative heading (Fig. 2C) in unclassified flights, which indicates that even bats outside mutual echolocation range avoid head-on flights.

In contrast to other species, where alignment occurs by copying the heading of the individual in front, e.g. in Surf scoters [38], we observed several coordinated flights where the reactor was in front of the actor (Fig. 2A and 2D when ∣*θ*_*ij*_∣ > 90°). Although our dataset is limited in size, these large exposure angles indicate that bats may be able to respond to an individual behind them because echolocation can provide information from rear angles e.g. in Fig. 2A an interaction, represented by a row of green points, tracing just outside the -40 dB contour from a position of 3 o’clock (lateral) to 6 o’clock (directly behind). Also note that in contrast to other movement coordination studies foraging Daubenton’s bats search for ephemeral and depletable prey and therefore their direction of motion is not targeted at one specific location of a shareable resource. As a result their respective position and orientation could change considerably facilitating actor-reactor swaps within an interaction.

Our BSMI model was able to create interactive behaviours (chases and coordinated flights) given that the model is based on biosonar perception with no input from movement interaction data other than observed flight speeds and turning angle distributions. Most importantly, the model output can reproduce the observed spatial interaction patterns only when the emission directionality *A*, hearing threshold *B* and delay were at their natural values. The best fit directionality value *A* = 16 is in the middle of the known range of emission directionality for this species (*A* = 7.3 for an independent measurement shown in S4 Fig., and *A* = 25.7 [29]), and the hearing threshold of *B* = 10 dB is also within the expected range [34].

In this study we have provided an answer to *how* individuals react to each other while they are foraging together, but it was not possible to determine *why*. As at least some data were collected at dates after juvenile bats had taken flight, it is not entirely impossible that some flight interactions were training flights between mothers and their offspring. Increased foraging success might be a driver for coordinating flight manoeuvres. This might be possible because bats that coordinate their movement at short distance have each other survey zones overlap and may thus enter into coordinated exploitations in presence of locally rich prey patches. Alternatively, with small prey density a bat may enter agonistic interactions forcing conspecifics away to monopolise a diminishing resource patch. The chases we observed might well contain such an agonistic component [39]. With the help of collective movement models of delayed interaction [40] future research could address some of these issues by testing responses of conspecifics to the locations and timings of foraging success of others, which they inadvertently indicate by their feeding buzzes.

In conclusion, we corroborate our BSMI hypothesis of foraging bats by (i) classifying bat interactions in the wild from highly resolved trajectory data, (ii) explaining the individuals’ relative positions based on the calculated biosonar perception field, and (iii) fitting the experimental observations with a bio-inspired movement model of interacting bats. In the process we have constructed a procedure to identify actor and reactor based not on their relative distance but on the delay dynamics of their relative orientation (defined through relative headings and exposure angles), which is independent of the animal species studied and can be applied to all types of coordinated movement data. The emerging actor-reactor relations and the associated delays with which an animal copies the heading of a conspecific provides a moving reference frame with which to determine when, where and how individuals respond to the actions of a conspecific and represents a powerful tool in the arsenal of computational techniques for movement ecology [41, 42].

## Methods

### Experimental methods

Animal movement trajectories were obtained by videogrammetry using two 525EX CCIR (Watec Inc., Newburgh, NY) with Cinegon 1.8/4.8 IR lens (Jos. Schneider Optische Werke GmbH, Bad Kreuznach, Germany) on tripods at heights between 4.13 and 5.66 m above the water surface as measured with Laser rangefinder DLE50 professional (Robert Bosch GmbH, Gerlingen, Germany). Pitch towards the water surface and lateral roll angles of the cameras were measured using Clinometer GeoMaxiclin (Geo Supplies Ltd, Sheffield, UK) to the nearest half degree. The recording area of approximately 900 m^2^ was illuminated with 850 nm IR light by a Raymax 200 (Raytec, Ashington, UK) positioned between the cameras at the shoreline. The video outputs were recorded synchronously on one XM-DVR PRO S153 (Datatoys, Mequon, MI) solid state dual-channel video recorder. Videos with bat flights were selected manually. Bat positions in the videos were selected by hand in every frame and the corresponding spatial positions calculated in custom written scripts (M. Holderied and H. Goerlitz) in MATLAB (The MathWorks Inc., Natick,MA). Each camera was calibrated and pixel based ray bundles for each camera were obtained using VMS software (Mark Shortis and Stuart Robson). Fields of view of both cameras did overlap only slightly and bat positions were not triangulated by stereo-imaging but rather by intersection of the pixel bundles originating at position, height and orientation of the single respective camera with a 2D plane 20 cm above the water surface, which was taken as the average flight height of this species [43]. The slight left-right asymmetry in the dataset indicates a preference for clockwise turns and a slight tendency to move parallel to the shoreline (S5 Fig.). This resulted in fewer trajectories moving away from the cameras, and thus making the (projected) reconstruction of the flight paths with similar level of errors. Videos were recorded in VGA format at 25 Hz frame rate. Each VGA frame is based on two half frames (odd and even lines taken in alternation). We analysed each half frame independently and interpolated the respectively missing odd or even lines such that the effective rate was 50 half frames per second, allowing for a temporal resolution of flight tracking equal to Δ*t* = 20 ms. This is about a factor four higher than the average call interval, i.e. the information update rate of Daubenton’s bats [32].

### Data segmentation

The dataset of paired bat trajectories was analysed to determine the fraction of data where interactions had occurred. For those data deemed to represent interaction we identified leader and follower dynamics in pairs of co-flying bats based on the sign of the automatically extracted delay values as a function of time with the help of the time-dependent directional correlation (TDDC) function, shown in Equation (2) below, and the time-dependent distance separation (TDDS) function, displayed below in Equation (4). Analysis of the TDDC and TDDS plots allowed us to segment paired movement trajectories into different behaviours.

#### The time-dependent delayed directional correlation

A good indicator of the alignment of two individuals is the normalised scalar product of the velocity of bat *i* at time *t* with bat *j* at time *t* + *τ* expressed by

$$\begin{array}{c} \nu_{ij}\left(t,\tau \right)=\frac{{\vec{v}}_{i}\left(t\right)}{|{\vec{v}}_{i}\left(t\right)|}\cdot \frac{{\vec{v}}_{j}\left(t+\tau \right)}{|{\vec{v}}_{j}\left(t+\tau \right)|}, \end{array}$$ (1)

which varies between 1, indicating perfect alignment, 0, when the heading of the bats is orthogonal, and -1 when in opposite directions.

In our recorded trajectories it was clear that bats changed behaviour dynamically as time *t* progressed. Integrating *ν*_*ij*_(*t*, *τ*) over all *N* values of the observation times [6] would wash out many of the transient features. An average over a smaller time-window empirically adjusted to the temporal scale of the observed dynamics was more appropriate [7, 44, 45] and represented a time-averaged correlation between the direction of the two bats. This averaging was also necessary to compensate for high-frequency noise due to pixelation and measurement errors. For each paired trajectory we thus computed the TDDC function

$$\begin{array}{c} C_{ij}\left(t,\tau \right)=\frac{1}{2w+1}\sum_{k=-w}^{w}\nu_{ij}\left(t+t_{k},\tau \right), \end{array}$$ (2)

where *v*_*ij*_(*t*, *τ*) has been averaged over a symmetric time-window 2*w*Δ*t* (*t*_*k*_ = *k*Δ*t*) for times *t* and delays *τ* constrained by

$$\begin{array}{ccc} w\;\Delta t< & t & <(N-w)\Delta t,\\ -t\leq & \tau & \leq N\Delta t. \end{array}$$ (3)

These constraints generate the sharp boundaries (see e.g. in Fig. 1B, S1 Fig. panel (b) and S2 Fig. panels (c) and (e)) and correspond to the line of points given by *τ* + *t* = 0 s and *τ* + *t* − *N*Δ*t* with Δ*t* = 0.02 s. To obtain sufficiently smooth trajectories the window width *w* = 2 was chosen, corresponding to a moving average over five data points for each coordinate, two on each side of the spatial coordinate pair. Note that the horizontal axis is relative to bat *i*. To interpret time relative to bat *j* one ought to compute *C*_*ji*_(*t*, *τ*), but only the exchange *i* → *j* together with *t* → *t* − *τ* in *C*_*ij*_(*t*, *τ*) would produce the same TDDC plot.

To define when two animals performed an interacting flight, it was necessary to set a threshold value *v*_*c*_ for *C*_*ij*_(*t*, *τ*) at and above which the heading of the two bats were deemed sufficiently correlated. To determine the effects of the choice of correlation threshold on our results, we studied how the extracted delays change as a function of *v*_*c*_. Since the appropriate *v*_*c*_ is clearly species dependent, requiring a judgement of an animal’s ability to align, our choice was a compromise guided by the need to capture bats’ coordination in the observed manoeuvres, e.g. the turns displayed in Fig. 1A. On one hand, we used a sufficiently high threshold to avoid misinterpreting noise features with alignment and to capture when a reacting animal attempted to copy the actions of the actor. On the other hand, we reduced the threshold value from *v*_*c*_ = 1, corresponding to persistent and perfect alignment, to increase the number and length of the interacting trajectories across the entire dataset. In S6 Fig. we display how the length of these interacting segments changes in each trajectory for different choice of *v*_*c*_. Overall there is a limited increase on the number of interacting data points for threshold values smaller than 0.95. In addition Gaussian fit of the resulting delay histograms for values 0.85 ≤ *v*_*c*_ ≤ 0.95 did not show significant differences either in the standard deviation or the 95% confidence interval from a Gaussian fit of Fig. 1C with the choice *v*_*c*_ = 0.95. The choice *v*_*c*_ = 0.95 was hence used to identify contour levels in the TDDC plot where interactions might have occurred (e.g. in Fig. 1B, S1 Fig. panel (b), S2 Figs. panels (c) and (d)).

#### The time-dependent delayed separation

To identify chase flight behaviour in the dataset, we considered *R*_*ij*_(*t*, *τ*), the spatial separation between bat *i* at time *t* and bat *j* at time *t* + *τ* averaged over a time-window *w*, namely

$$R_{ij}\left(t,\tau \right)\;=\;\frac{1}{2w+1}\overset{w}{\underset{k=-w}{\sum^{\text{​}}}}{\left\{{\left[x_{i}\left(t+t_{k}\right)-x_{j}\left(t+\tau +t_{k}\right)\right]}^{2}++{\left[y_{i}\left(t+t_{k}\right)-y_{j}\left(t+\tau +t_{k}\right)\right]}^{2}\right\}}^{1/2}.$$ (4)

In Equation (4) (*x*_*l*_(*t*), *y*_*l*_(*t*)) with *l* = *i* or *j* is the coordinate pair of bat *i* or *j* at time *t*. When this separation equals zero for positive *τ*, then bat *j* occupied all previous positions that bat *i* had between time *t* − *τ* − 2Δ*t* and *t* − *τ* + 2Δ*t*. To avoid classifying trajectories where bats crossed each other’s paths with arbitrary headings and delays as chases, the set of paired values (*t*, *τ*) where interaction occurred were first extracted from the analysis of *C*_*ij*_(*t*, *τ*). *R*_*ij*_(*t*, *τ*) was then computed at the specific (*t*, *τ*) values and the interaction was classified as chase if *R*_*ij*_(*t*, *τ*) ≤ *d*_*c*_. Hence chase flights were a sub-category of coordinated flights, as bats were both well aligned, *C*_*ij*_(*t*, *τ*) ≥ *v*_*c*_, and had small spatial separation, *R*_*ij*_(*t*, *τ*) ≤ *d*_*c*_, in the delayed reference frame. The threshold used was *d*_*c*_ = 0.15 m, which is smaller than 0.25 m, the average wingspan of a Daubenton’s bat. It turned out that for all behaviours classified as chases the average separation in delayed time were at most 0.09 m.

#### Extracting delay values

For each time *t* in the TDDC and TDDS plots within the respective regions of interaction, i.e. *C*_*ij*_(*t*, *τ*) ≥ *v*_*c*_ and *R*_*ij*_(*t*, *τ*) ≤ *d*_*c*_, it is possible to associate different delay values *τ*. A procedure was thus developed to extract a single interaction delay *τ*, and its associated uncertainty, for each time *t*. These values can then be used to identify leader and follower and subsequently to classify the trajectory in terms of the behavioural mode of interaction (chase and coordinated flight).

There are several potential methods to extract such ‘interaction path’ (delays as a function of time) from the TDDC plot. Two straightforward methods consist of taking the delay value which maximises the correlation: over the extent of the entire recording of a bat pair [6], or over a time-window, either forward from [44, 45] or symmetric around each time-step [7]. Whilst easy to perform, these procedures are poor at reconstructing biologically realistic interactions most importantly because they violate the simple assumption that each bat’s reactions are strictly ordered in time. To understand this, consider that bat *i* acts at time *t* and bat *j* reacts at time (*t* + *τ*) then a subsequent action by bat *i* at time *t* + Δ*t* can be reacted to by bat *j* at time (*t* + Δ*t* + *τ* + Δ*τ*), where Δ*τ* represents a change in delay. If Δ*τ* < −Δ*t* then (*t* + Δ*t* + *τ* + Δ*τ*) < (*t* + *τ*), and bat *j* performed the second reaction before the first reaction at time (*t* + *τ*). Such situations are deemed nonsensical and hence changes in delay should be restricted to Δ*τ*/Δ*t* ≥ −1.

In the TDDC plot all possible ‘interaction paths’ represent delay values that change over time. The actual response delay across the trajectory is obtained by computing the cumulative value of *C*_*ij*_(*t*, *τ*) from left to right, i.e. from the first to the last observation time, according to three requirements: *C*_*ij*_(*t*, *τ*) < *v*_*c*_, the change of delay along a path must fulfil the time-ordered requirement, and the actual *C*_*ij*_(*t*, *τ*) value along a possible path gets reduced if delay changes over time.

More specifically, each possible path starts from the coordinates (*t*_0_, τ*), with *t*_0_ = *w*Δ*t* and (−*N* + *w*)Δ*t* < τ* < (*N* − *w*)Δ*t*, and finish at (*t*_max_, *τ*_max_), with *t*_max_ = (*N* − *w*)Δ*t*. Any path is thus represented by the coupled elements (***t***, ***τ***) with

$$\begin{array}{ccc} t & = & \left\{t_{0},t_{1},t_{2},\ldots ,t_{\text{max}}\right\} \end{array}$$ (5)

and the corresponding delay values,

$$\begin{array}{c} \tau =\left\{\begin{array}{c} {\vec{\tau }}^{0}\\ {\vec{\tau }}^{1}\\ \ldots \\ {\vec{\tau }}^{M} \end{array}\right\}=\left\{\begin{array}{ccccc} \tau_{0}^{0}, & \tau_{1}^{0}, & \tau_{2}^{0}, & \ldots , & \tau_{\text{max}}^{0}\\ \tau_{0}^{1}, & \tau_{1}^{1}, & \tau_{2}^{1}, & \ldots , & \tau_{\text{max}}^{1}\\ \ldots & & & & \\ \tau_{0}^{M}, & \tau_{1}^{M}, & \tau_{2}^{M}, & \ldots , & \tau_{\text{max}}^{M} \end{array}\right\}, \end{array}$$ (6)

with *M* limited by the various constraints.

The ‘optimal’ path has the highest overall correlation for each pairwise trajectory given by

$$\begin{array}{c} p_{l}\left({\vec{\tau }}^{\;l}\right)=\sum_{n=w}^{N-w}\left\{\begin{array}{cc} C_{ij}\left(t_{n},\tau_{n}^{\;l}\right), & \text{if}\;\;\;C_{ij}\left(t_{n},\tau_{n}^{\;l}\right)\geq v_{c}\;\;\text{and}\;\tau_{n}^{\;l}=\tau_{n-1}^{\;l},\\ C_{ij}\left(t_{n},\tau_{n}^{\;l}\right)-q, & \text{if}\;\;\;C_{ij}\left(t_{n},\tau_{n}^{\;l}\right)\geq v_{c}\;\;\text{and}\;\tau_{n}^{\;l}\neq \tau_{n-1}^{\;l},\\ 0 & \text{otherwise,}\; \end{array}\right. \end{array}$$ (7)

where *q* is a penalising cost associated with changing delay. The inclusion of this penalising cost in the algorithm for extracting delay values was imposed to account for the added cognitive requirements that an animal would incur if it were to interact by constantly changing the timeframe of reference. As the animals’ relative locations fluctuate considerably during an interaction—change of speed and heading is a necessity during foraging activities—a constant delay may in fact represent a steady source of information through which to control the conspecific’s actions and take decisions accordingly. The appropriate *q* value to use depends on the profile of the correlation map which in turn is affected by the spatio-temporal trajectories. For our bats, the value of *q* had to be small enough to allow for delay changes when approaching the contours delimiting the interaction region in the correlation map, and large enough to restrict the change of delays when away from those limiting contours. These goals were met with the choice *q* = 0.02.

By setting to 0 the contributions of all paths with profiles Δ*τ*/Δ*t* ≥ −1 and favouring those with zero slope, the extracted delays represent a compromise between having the longest segments within the disconnected regions of interaction and the shallowest slope. In this way we identify interaction events as those behaviours with the longest duration and with limited variations in delay response. In other words we associate delayed interactions with actions and reactions that are persistent rather than brief or instantaneous.

The procedure for extracting delays for chase flight behaviour differs slightly. Any region of the TDDC map exceeding the threshold *v*_*c*_ and with the corresponding TDDS region less than or equal to *d*_*c*_ pertains to chase-flight, therefore the interaction path is constructed by minimising the delayed separation, (rather than maximising the directional correlation), again satisfying the constraint that interactions be strictly ordered in time and with a penalising cost for changing delay. If a trajectory contains both chase flight and coordinated flight the chase flight interaction path is found first and then the coordinated flight interaction path is chosen as the maximum correlation path that connects to the chase paths.

#### Uncertainty in delay extraction

Current methodologies to determine reaction delays from movement data are unable to specify ranges of uncertainty of the extracted values. The use of the TDDC and TDDS, however, provides the means to quantify this delay uncertainty. This uncertainty, which can be asymmetric around the extracted delay, is affected by the classification (chase or coordinated flight) as well as the curvature of the trajectories with lower uncertainty being obtained when bats make interactive turns as compared to when they move straight in coordination.

Choosing between multiple delays at a given time in the region of high correlation in the TDDC plot is a source of uncertainty. For coordinated flight the number of possible delays at each time is dependent on the trajectories themselves. Consider two extreme examples, one where the paired trajectories are parallel lines and the other where both trajectories contain many turns and are therefore curved and winding. In the first case the bats are perfectly aligned at all times and for all delays. In such a case *C*_*ij*_(*t*, *τ*) ≥ *v*_*c*_ for all (*t*, *τ*), and identifying uniquely the delay with which the bats performed this flight is not possible. In the second case, as a result of all the turns, the delay with which the bats flew is more apparent and the number of delays that exceed the correlation threshold will converge to the correct one as the threshold is increased.

For a given threshold, one could determine the range of values around the extracted delay for each value of time in the TDDC plot. However using the relationship between the delay range and the curvature of the trajectory allows to obtain a smaller bound of the delay uncertainty. To understand this, consider S7 Fig. where we plot an hypothetical TDDC function whereby the associated paired trajectories contain flight segments with high-curvature ending at time *t*_*hc*_. These segments subsequently transition to parallel motion for both individuals beginning at time *t*_*p*_. There is obviously a large range of possible delays for the parallel segment, $\left[\tau_{p}^{↓},\tau_{p}^{↑}\right]$, and a small range, $\left[\tau_{hc}^{↓},\tau_{hc}^{↑}\right]$, for the high-curvature segment, and a highly dynamic range for the transition region *t*_*hc*_ ≤ *t* ≤ *t*_*p*_. However due to our condition, Δ*τ*/Δ*t* ≥ −1, if the change in the minimum delay is greater than the elapsed time, i.e. $(\tau_{p}^{↓}-\tau_{hc}^{↓})/(t_{p}-t_{hc})<-1$, (or similarly for the maximum delays looking backwards in time), some of the parallel-flight delays cannot contribute to the interaction path and the delay choice is made from the reduced range, $\left[\tau_{p*}^{↓},\tau_{p}^{↑}\right]$. In this way knowledge from the high-curvature flight has reduced the uncertainty in choosing delays from the parallel-flight. Although we have only compared two times to illustrate the idea, the procedure consists of drawing the −1 gradient lines forward in time at each time point from the minimum delay value, and backward in time from the maximum delay value. Anywhere these gradient lines are within a threshold contour, the delay is reduced to the value along that line. For example for all times between the marked values of *t*_1_ and *t*_2_, the −1 gradient line lies inside the contour and so reduces the delay-range for those times. The extracted delay uncertainty is therefore quantified as the delay range after the time ordered condition has been applied to each point along the contour.

For coordinated flights we thus have that the straighter a trajectory, the larger is the uncertainty in the delay. This however does not occur in chase flights for which the delay uncertainty is independent of the curvature of the flight because only a small delay window allows one bat to occupy the other bat’s previous position (see e.g. S1 Fig. panel (d) and S2 Fig. panel (f)). Hence the uncertainty in the chase flight delays is quantified simply by the (small) range of possible delays.

A comparison between the use of the time-independent directional correlation function [6], as well as of the ‘maximal’ path in *C*_*ij*_(*t*, *τ*) [7], with our procedure to extract delay values is displayed in the 8-panel S8 Fig. In panels (d) and (f) one can see that tracking the maximum of the TDDC function gives a delay path that displays sudden jumps, at times between positive and negative values. Furthermore, it is apparent from panels (g) and (h) that this maximum delay path violates our time-ordering procedure being outside the allowed uncertainty. A clear example of the inability to identify the correct response delays by tracking the maximum of the directional correlation is shown in S8 Fig. panel (f). There, around *t* = 1.3 s the maximum of *C*_*ij*_(*t*, *τ*) (green line) suddenly jumps from *τ* ≃ 0.2 s to *τ* = 1 s. This jump occurs because bat *i*’s heading after the right turn at *t* = 1.4 s is approximately parallel to bat *j*’s heading after its own right turn at *t* = 2.3 s (see the corresponding trajectory plot in Fig. 1A).

### The BSMI model

An agent based model was created to demonstrate the effects of asymmetric perception and reaction delays on the spatio-temporal trajectories of simulated bat pairs. The model contains the minimal ingredients necessary to account for the salient sensory biology features of the bats and their interaction. The individual movement statistics of a bat follows that of a correlated random walker [46, 47] with turning angles and speed values obtained from the observed ones. Each animal is limited in its manoeuvrability by aerodynamic constraints and tries to align to a conspecific present within its interaction field. Each bat echolocates every 100 ms with a certain hearing and emission directionality and detects the presence of the other animal only when above a certain threshold. When another bat is detected, an individual responds by copying the other’s heading with some delay (see Equations (8–12) and S9 Fig. for further details). The simulations were run in discrete time, for 10 s and Δ*t* = 20 ms corresponding to the resolution of the observations, and continuous space by generating initial random positions and headings inside a region of 30 × 30 m^2^.

#### Flight dynamics

Bat *i*’s heading *ϕ*_*i*_(*t*), speed magnitude *v*_*i*_(*t*) and location (*x*_*i*_(*t*), *y*_*i*_(*t*)) are updated at each time step as follows:

$$\begin{array}{ccc} v_{i}\left(t\right) & ∼ & 𝓡(4.81\;\text{m}\;{\text{s}}^{-1},2.18\;\text{m}\;{\text{s}}^{-1}),\\ \phi_{i}\left(t\right) & = & \left\{\begin{array}{cc} \gamma_{i}\left(t\right), & \text{if}\;|\gamma_{i}\left(t\right)-\phi_{i}\left(t-\Delta t\right)|\leq \beta_{i}\left(t\right)\Delta t,\\ \phi_{i}\left(t-\Delta t\right)+\beta_{i}\left(t\right)\Delta t, & \text{if}\;\gamma_{i}\left(t\right)-\phi_{i}\left(t-\Delta t\right)>\beta_{i}\left(t\right)\Delta t,\\ \phi_{i}\left(t-\Delta t\right)-\beta_{i}\left(t\right)\Delta t, & \text{if}\;\gamma_{i}\left(t\right)-\phi_{i}\left(t-\Delta t\right)<\beta_{i}\left(t\right)\Delta t, \end{array}\right.\\ x_{i}\left(t\right) & = & x_{i}\left(t-\Delta t\right)+v_{i}\left(t\right)\;\Delta t\cos\left[\phi_{i}\left(t\right)\right],\\ y_{i}\left(t\right) & = & y_{i}\left(t-\Delta t\right)+v_{i}\left(t\right)\;\Delta t\sin\left[\phi_{i}\left(t\right)\right]. \end{array}$$ (8)

In Equation (8) the symbol ∼ means that *v*_*i*_(*t*) is drawn from the Rician distribution [48] 𝓡(*μ*, *σ*) with non-centrality parameter *μ* and scale parameter *σ*, which was fitted (*R* ^2^ = 0.97) to the observed movement patterns of paired flying bats. The desired heading of bat *i*, that is the heading that a bat would choose depending only on its behavioural state (see below) at time *t*, is *γ*_*i*_(*t*). The actual heading at time *t* − Δ*t* and time *t*, taken by bat *i* once the aerodynamic constraints have also been accounted for, are represented, respectively, by *ϕ*_*i*_(*t* − Δ*t*) and *ϕ*_*i*_(*t*). The angular rate of change *β*_*i*_(*t*) is limited by aerodynamic constraints corresponding to a maximum lateral acceleration *a*_*c*_ of 4 g (4 times the gravitational acceleration of 9.81 ms^−2^), providing a maximum given by *β*_*i*_(*t*) = *a*_*c*_/*v*_*i*_(*t*). The 4 g limit was measured to contain 99.9% of the turning angles from the paired flight data. Examples of an interacting and independent path generated by the BSMI model are displayed in S9 Fig.

The bat may be in either of two behavioural states: independent or interacting. The behavioral state determines the shape of the distribution from which an individual would draw its desired turning angle *γ*_*i*_(*t*), and it is updated in two ways. (i) Every five time steps an individual would send an echolocation call to determine the heading of the other bat. When a conspecific is detected, a bat enters an interacting behavioural state and attempts to align by selecting a turning angle that reduces the difference in relative heading. If the other bat is not detected, the individual enters an independent state and it maintains that behaviour until it detects a conspecific. Once in the independent state the bat selects a turning angle from a broader distribution. (ii) The other way a bat may change its behavioural state is by moving from the interacting to the independent state at each time step. Even though a bat is in the process of aligning to another individual detected in the last echolocation call, it may find convenient to evade alignment, e.g. for the purpose of being the first to detect a prey or to ensure exclusive access to certain areas of the water’s surface. To reproduce this feature in the model an individual in the interaction state may choose, at each 20 ms time step, to enter an independent state with a small probability of 0.05.

#### Independent flight

When an individual is in the independent state, the animal’s turning angles are drawn from a von Mises distribution [49], displayed in S9 Fig. panel (a), centred around its heading at the previous time step. The desired heading of bat *i* at time *t* is thus

$$\begin{array}{c} \gamma_{i}\left(t\right)∼𝓜\left(\phi_{i}\left(t-\Delta t\right),557\right), \end{array}$$ (9)

where 𝓜(*ξ*, *κ*) represents a von Mises distribution with mean direction *ξ* and concentration parameter *κ*. The concentration parameter *κ* = 557, was obtained by a fit (*R* ^2^ = 0.997) to the turning angles of unclassified flying bats.

#### Interacting flight

lnteraction in the form of alignment was constructed by setting a bat’s desired heading as the mean of its own with the one of its conspecific [2, 50] but with the characteristic feature that the heading of the conspecific is the one at time *τ* in the past. In other words, at time *t* the desired heading of bat *i* is drawn from a von Mises distribution centred around the average between bat *i*’s heading at time *t* − Δ*t* and the heading that bat *j* had at time *τ* in the past. The concentration parameter of the von Mises distribution was chosen to be intermediate between the turning angle distribution observed in unclassified flights and a distribution with no noise. To avoid the need to impose additional assumptions that specify under what conditions individuals would choose to enter a coordinated or chase behaviour, we used the turning angle distribution fitted (*R* ^2^ = 0.999) to the observed chases, whose concentration parameter was *κ* = 2473 (S9 Fig. panel (b)). The desired heading of bat *i* is thus

$$\begin{array}{c} \gamma_{i}\left(t\right)∼𝓜\left(\frac{\phi_{i}\left(t-\Delta t\right)+\phi_{j}\left(t-\Delta t-\tau \right)}{2},2473\right). \end{array}$$ (10)

### Sound field modelling

#### Echolocation directionality

A bat may enter into an interacting state if the received echo amplitude is above its hearing threshold. This echo amplitude is the result of the echolocation directionality of the animal as well as the target strength of the echo-giving object and the transmission losses during sound propagation [51]. By considering a source level SL = 110 dB [29] and a target strength TS = -10 dB, the echo amplitude *α*, expressed in dB, at angle *ζ* from the emission direction and returning from a bat at distance *ρ* in meters, is given by

$$\begin{array}{c} \alpha \left(\rho ,\zeta \right)=\text{SL}+\text{TS}+\left\{\left(A+2\right)\left[\cos\left(\zeta \right)-1\right]\right\}+2\left[20\log\left(\frac{0.1}{\rho }\right)+\rho c\right]. \end{array}$$ (11)

The third term, in curly brackets, in Equation (11) corresponds to the azimuthal echolocation directionality of the bat consisting of (i) an emission directionality with a (positive) asymmetry parameter *A* and (ii) a hearing directionality. The representation of the emission directionality with a cosine law is supported by the experimental results displayed in S4 Fig. Hearing directionality was calculated from a head-related transfer function (HRTF) obtained from a 3D acoustic modelling of a *Myotis daubentonii* head for the frontal hemisphere and for frequencies between 35 and 45 kHz [52]. Hearing directionality was very broad with attenuation being -2 and -3 dB at ±90°, respectively. For simplicity we also fitted a cosine function giving an asymmetry parameter equal to 2 dB (*R* ^2^ = 0.44), which we have used in the third term of Equation (11). The fourth term, in square parenthesis, is (i) the sound attenuation of a spherical point source relative to a reference distance of 0.1 m, and (ii) the sound absorption losses *c* = −1.28 dB m^−1^ for 10°C, 86% relative humidity and 45 kHz following [22]. The factor of 2 in front of the square parenthesis accounts for the distance to the target and back.

#### Flight dynamics simulation with echolocation

In a simulation run of the model, every five time steps bats *i* and *j* emit an echolocation call with the axis of emission corresponding to the bats’ headings. The attenuation of the emitted sound at the location of the other bat is then estimated by knowing the exposure angles *θ*_*ij*_(*t*) and *θ*_*ji*_(*t*) and their relative distance *d*(*t*) = *d*_*ij*_(*t*) = *d*_*ji*_(*t*), and computing *α*_*ij*_(*d*(*t*), *θ*_*ij*_(*t*)) for the sound emitted by bat *i* and *α*_*ji*_(*d*(*t*), *θ*_*ji*_(*t*)) for the sound emitted by bat *j*. If *α*_*ij*_ or *α*_*ji*_ are greater than the attenuation threshold *B*, the bats carry on independently of the conspecific. However if either attenuation is less than or equal to *B* the corresponding bat will enter the interacting state after a reaction delay *τ*. Combining Equation (9) and (10), the desired heading of bat *i* becomes

$$\begin{array}{ccc} \gamma_{i}\left(t\right) & ∼ & \left\{\begin{array}{cc} 𝓜(\phi_{i}\left(t-\Delta t\right),557) & \text{if}\;\alpha_{ij}\left(d\left(t-\tau \right),\theta_{ij}\left(t-\tau \right)\right)>B\\ 𝓜\left(\frac{\phi_{i}\left(t-\Delta t\right)+\phi_{j}\left(t-\Delta t-\tau \right)}{2},2473\right) & \text{if}\;\alpha_{ij}\left(d\left(t-\tau \right),\theta_{ij}\left(t-\tau \right)\right)\leq B \end{array}\right. \end{array}$$ (12)

and similarly for bat *j*.

To verify that the delays extracted from the movement trajectories are biologically meaningful, we ran the model using no delay, with delays within the observed range 100–500 ms, as well as larger delay values between 500 ms and 2 s. We generated spatio-temporal trajectories of co-flying pairs as a function of the emission directionality *A* and threshold *B* parameters with values -60 dB ≤ *B* ≤ 60 dB and 0 ≤ *A* ≤ 72. The model-generated trajectories were then analysed and classified with the analysis of the TDDC and TDDS plot, and the locations of the actor relative to the reactor compared to the observed spatial patterns of interaction as displayed in the relative position plot (Fig. 3).

## Supporting Information

S1 Video Sample spatio-temporal paired trajectory. Example of a paired flight trajectory, with 20 ms temporal resolution, corresponding to Fig. 1A.
(MOV)

S1 Fig Summary of the data analysis procedure. Graphical representation of the sequential steps involved in extracting behavioural states to identify actors and reactors (a-f) and their relative positions from a sample movement trajectory (g).
(TIFF)

S2 Fig Representative sample of coordinated flight and chase. The top panels show two different recorded trajectories from the dataset: (a) is an example of coordinated flight behaviour and (b) is an example of chase flight behaviour. Panels (c) and (d) show contour level plots of the corresponding TDDC function *C*_*ij*_(*t*, *τ*). Similarly panel (e) and (f) show the corresponding TDDS function *R*_*ij*_(*t*, *τ*). In panels (a) and (b) the similarity in the TDDC plots is evident. It is possible to distinguish clearly the bats’ behaviour only when comparing their respective TDDS plots.
(TIFF)

S3 Fig Bats’ relative locations and calculated actor’s sound emission field. Symbols indicate positions of the reactor relative to the location (centred) and heading (upwards) of the actor. For behaviour deemed unclassified, the individual at the centre is picked at random for each pair. Parameters of emission directionality, source level and sound absorption are the same as in Fig. 2A. Red lines are isocontours of the emitted sound field.
(TIFF)

S4 Fig Perceptual bias of *Myotis daubentonii*. Foraging flight trajectory and cumulative emission directionality of *Myotis daubentonii* over approximately a 30 m diameter pool. (a) Top view and (b) lateral view of flight trajectory. Open circles are 126 positions of individual sound emissions as determined by acoustic tomography. Filled symbols represent the positions of recording microphones. (c) Measured emission directionality with 15° resolution based on calibrated microphone recordings, and taking transmission losses of sound on the way from the bat to the microphone for the temperature and humidity [22] at the time of recording into account. Flight direction is to the top. Emission amplitude expressed in dB is normalized to the highest observed value. Thin solid lines: maximum and minimum relative amplitude. Bold solid line: mean relative amplitude. Bold dotted line: cosine function fitted to relative emission amplitudes. *A* is the parameter of the fitted cosine function, namely *A*[cos(*ζ*) − 1], resulting in a front-rear amplitude difference of 14.6 dB.
(TIFF)

S5 Fig Observed bat headings. Two polar histograms showing all the recorded headings of the individually flying bats (*a*) and the paired flying bats (*b*) captured during the experiment. The line connecting the two recording cameras corresponds approximately to 180° and 0°. In both panels the measured headings, rather than being uniformly distributed, are biased towards mean angles. Computing the mean and standard deviations we obtain 349.4° and 169.4°, (±33.1°), for the individual flying bats and 348.0° and 168.0°, (±36.7°), for the paired flying bats. This bias might be caused by the bats trying to avoid the perimeter demarcating their foraging environment: one part along the shoreline where the cameras were mounted and the other part perpendicular to the latter and located just outside the recording region.
(TIFF)

S6 Fig Sensitivity to the choice of directional correlation threshold. For each of the 70 trajectories that comprise the paired flight dataset, the percentage of that trajectory deemed interacting flight behaviour is shown for different correlation thresholds *v*_*c*_. The effect of changing *v*_*c*_ is trajectory-dependent with almost no change in some cases, e.g. 14, and large variation in others, e.g. 56. An analysis of each of the 70 paired flights with thresholds smaller than the one used (0.85 ≤ *v*_*c*_ < 0.95) reveals that only four trajectories (4, 38, 53, 59) brings about interacting behaviour that is otherwise missed. In all the other trajectories, a lower threshold simply increases the length of the interacting paths by converting unclassified segments to classified ones at the beginning or the end of a segment already deemed interacting. The right panel shows the total fraction of interacting flight behaviour for all the data for different thresholds *v*_*c*_. Trajectory 17, 20, 40 and 64 were depicted, respectively, in S2 Fig. panels (a) and (b), and S8 Fig. panels (a) (and also S1 Fig. panel(a)) and (b), whereas trajectory 7, 29 and 56, plotted respectively in S10 Fig. panels (a), (c) and (e), were deemed outliers and their entire dataset were catalogued as unclassified behaviour.
(TIFF)

S7 Fig Uncertainty in delay extraction. A schematic correlation map demonstrating the effect of trajectory curvature on delay extraction. Within the plot the correlations are distinguished by those below a selected threshold (white-space) and those above the threshold (shaded area), separated by the correlation threshold itself (black-line). Two distinct flight behaviours are drawn: high correlation flight where the bats are well aligned but turn frequently, and parallel flight where the bats fly in straight lines. Times and delays have been marked to demonstrate the effect of the time ordering condition (Δ*τ*/Δ*t* ≥ −1) on the uncertainty of delay extraction.
(TIFF)

S8 Fig Comparison of delay extraction procedures. In panels (a) and (b) two sample trajectories are drawn with their corresponding TDDC functions in panels (d) and (f), respectively. The maximum of the TDDC function averaged over all times is shown in panel (c) and (e), respectively. In panel (d) and (f) we also show the delay path extracted from our time-ordered procedure (black line) and from selecting the so-called ‘maximal path’ [7], that is the maximum at each time of Equation (2). For panel (d) delay values (and uncertainties) for our time-ordered procedure between 1 s < *t* < 2 s are extracted by considering the TDDS plot (displayed in S1 Fig. panel (d)) when the interaction becomes a chase. A comparison of the delay values extracted with the different procedures from panel (a) and (b) is displayed, respectively, in panels (g) and (h) with shaded light red areas representing the uncertainty in the extracted delay from our combined analysis of the TDDC and TDDS function.
(TIFF)

S9 Fig Observed turning angle distribution and BSMI model sample trajectories. Panels (a) and (b) compare the distributions of turning angles for the observed unclassified and chase flights, respectively. A fit of the histograms with a von Mises distribution is also shown as well as the maximum lateral acceleration imposed on the model animals. Panels (c) and (d) show two examples of trajectories that emerge from the model in which the bats act independently and interactively. At the end of each trajectory we show the bats’ echolocation range obtained by selecting hearing threshold of 10 dB and an emission directionality with an asymmetric parameter *A* = 16 in Equation (11). Panels (e) and (f) show the bats’ headings corresponding to the coloured trajectories in (c) and (d), respectively.
(TIFF)

S10 Fig Outliers. The paired panels (a)-(b), (c)-(d) and (e)-(f) show respectively the trajectory and correlation maps of bat pairs whose interactive behavioural identification was discarded because the animals’ separation distance was at the limit of their hearing range.
(TIFF)
