# Supplementary figures and images for: Delayed Response and Biosonar Perception Explain Movement Coordination in Trawling Bats
Source: PLoS Comput Biol. 2015 Mar 26;11(3):e1004089. doi: 10.1371/journal.pcbi.1004089 (PMC4374978; doi:10.1371/journal.pcbi.1004089)

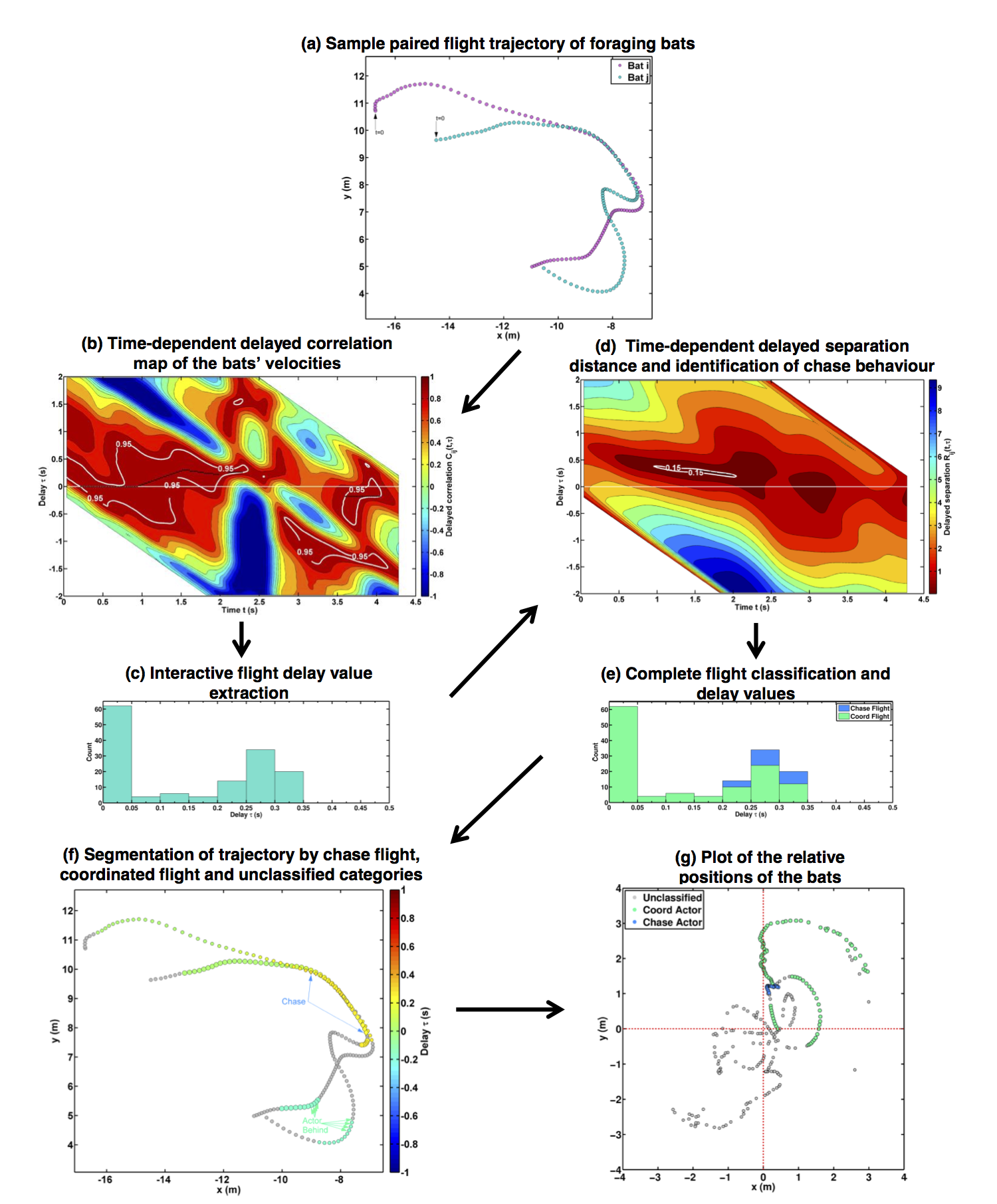

Supplement: S1 Fig — Graphical representation of the sequential steps involved in extracting behavioural states to identify actors and reactors (a-f) and their relative positions from a sample movement trajectory (g). (TIFF) [file pcbi.1004089.s002.tiff]

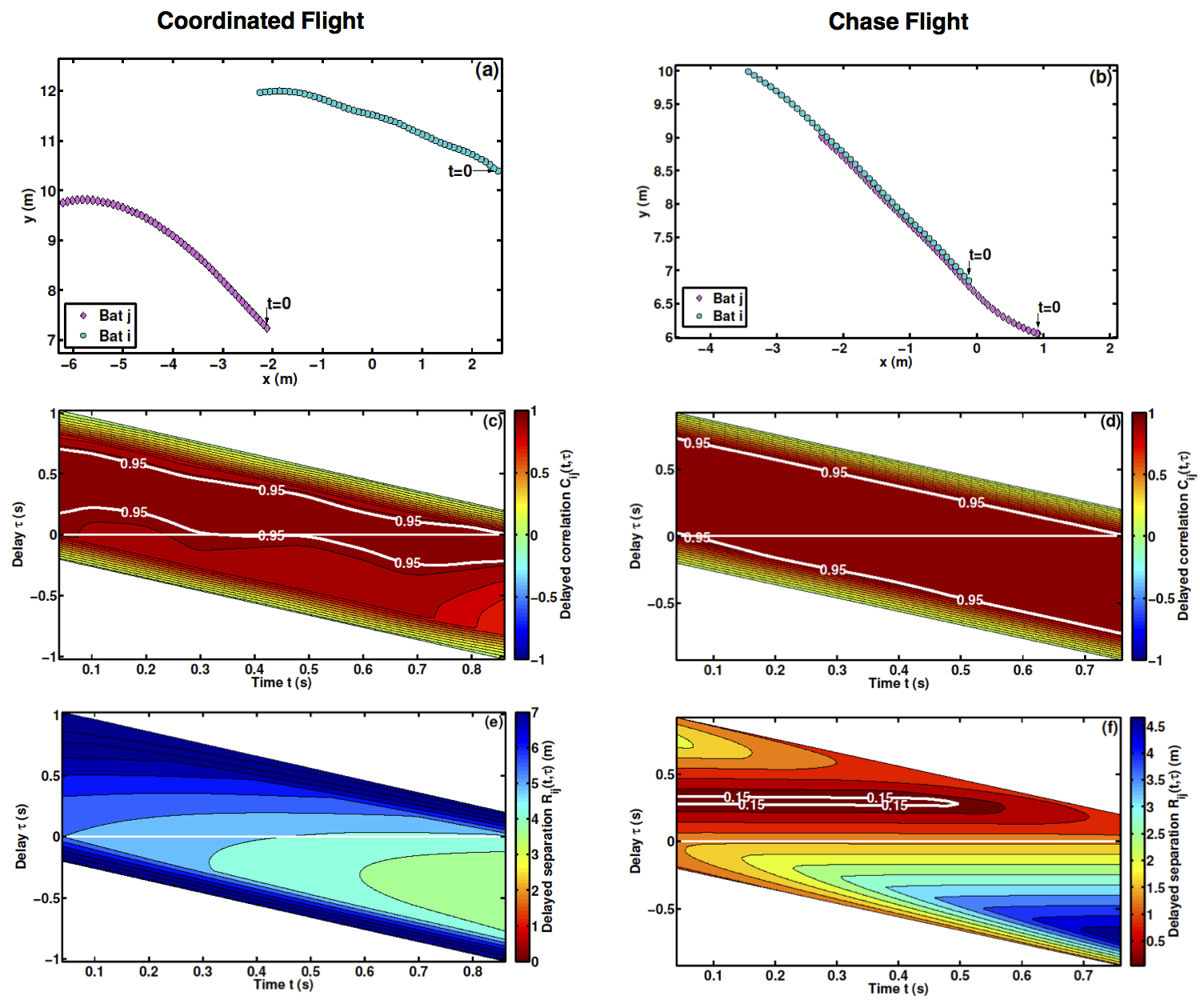

Supplement: S2 Fig — The top panels show two different recorded trajectories from the dataset: (a) is an example of coordinated flight behaviour and (b) is an example of chase flight behaviour. Panels (c) and (d) show contour level plots of the corresponding TDDC function C ij(t, τ). Similarly panel (e) and (f) show the corresponding TDDS function R ij(t, τ). In panels (a) and (b) the similarity in the TDDC plots is evident. It is possible to distinguish clearly the bats’ behaviour only when comparing their respective TDDS plots. (TIFF) [file pcbi.1004089.s003.tiff]

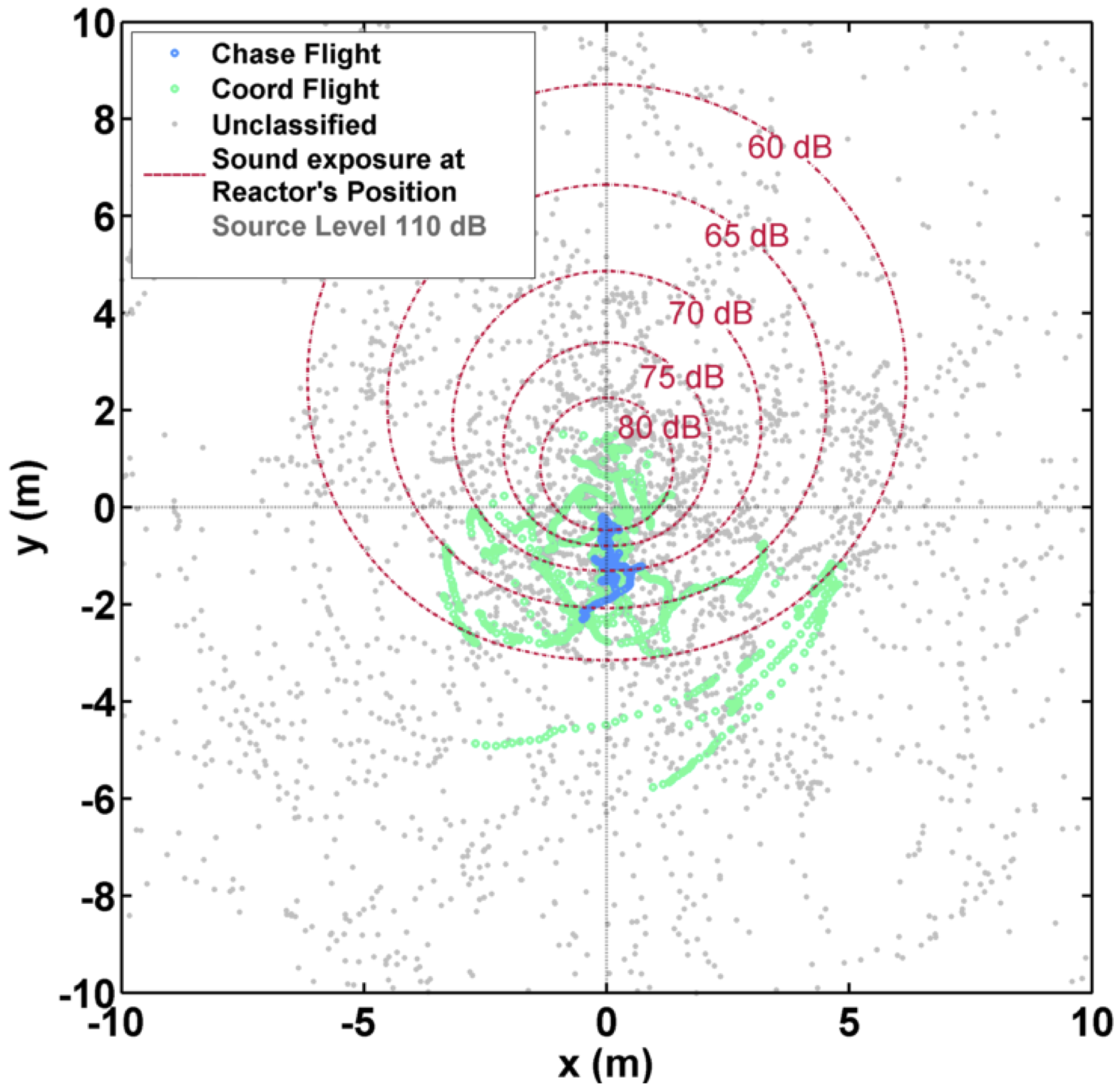

Supplement: S3 Fig — Symbols indicate positions of the reactor relative to the location (centred) and heading (upwards) of the actor. For behaviour deemed unclassified, the individual at the centre is picked at random for each pair. Parameters of emission directionality, source level and sound absorption are the same as in Fig. 2A. Red lines are isocontours of the emitted sound field. (TIFF) [file pcbi.1004089.s004.tiff]

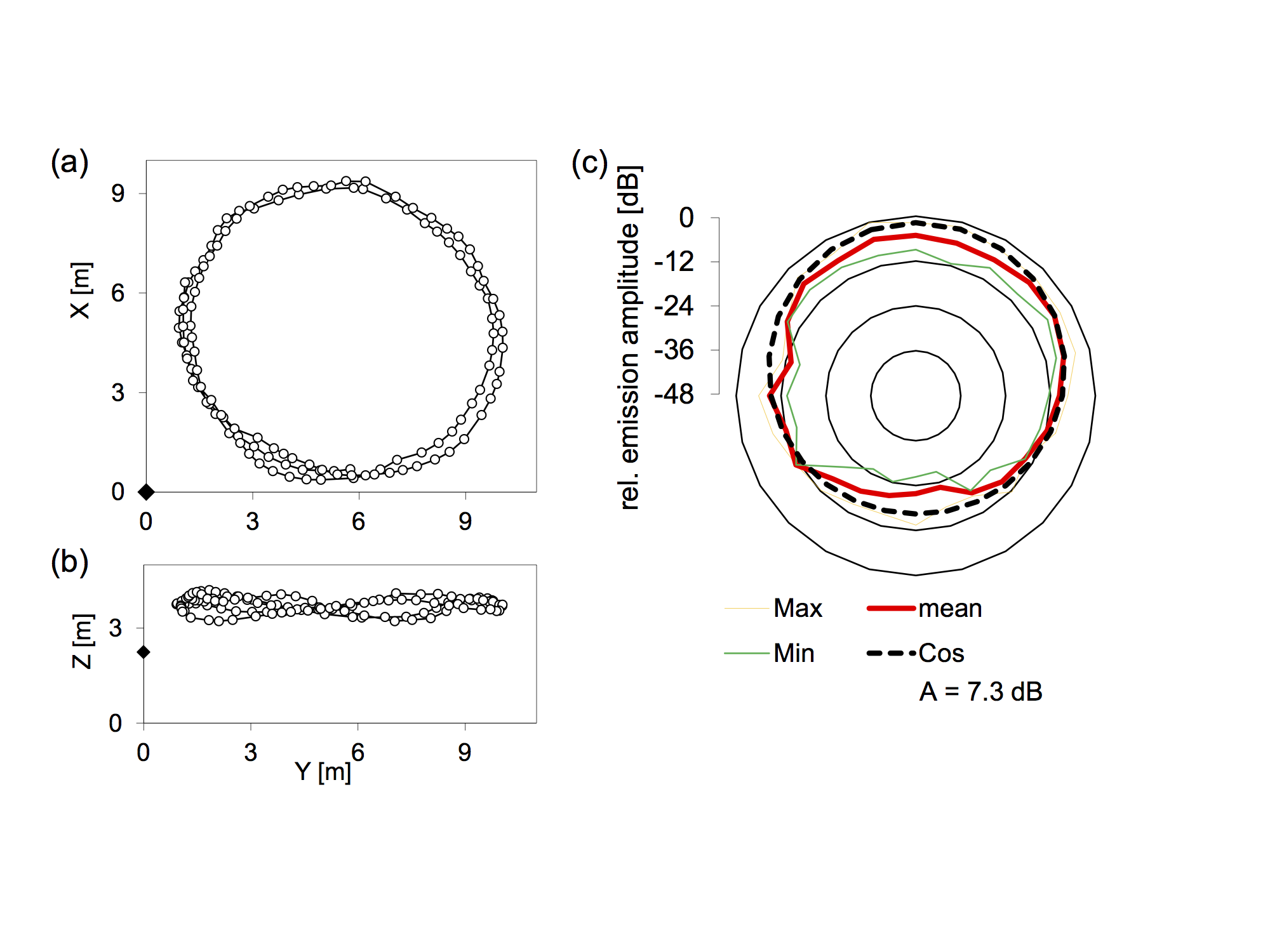

Supplement: S4 Fig — Foraging flight trajectory and cumulative emission directionality of Myotis daubentonii over approximately a 30 m diameter pool. (a) Top view and (b) lateral view of flight trajectory. Open circles are 126 positions of individual sound emissions as determined by acoustic tomography. Filled symbols represent the positions of recording microphones. (c) Measured emission directionality with 15° resolution based on calibrated microphone recordings, and taking transmission losses of sound on the way from the bat to the microphone for the temperature and humidity [22] at the time of recording into account. Flight direction is to the top. Emission amplitude expressed in dB is normalized to the highest observed value. Thin solid lines: maximum and minimum relative amplitude. Bold solid line: mean relative amplitude. Bold dotted line: cosine function fitted to relative emission amplitudes. A is the parameter of the fitted cosine function, namely A[cos(ζ) − 1], resulting in a front-rear amplitude difference of 14.6 dB. (TIFF) [file pcbi.1004089.s005.tiff]

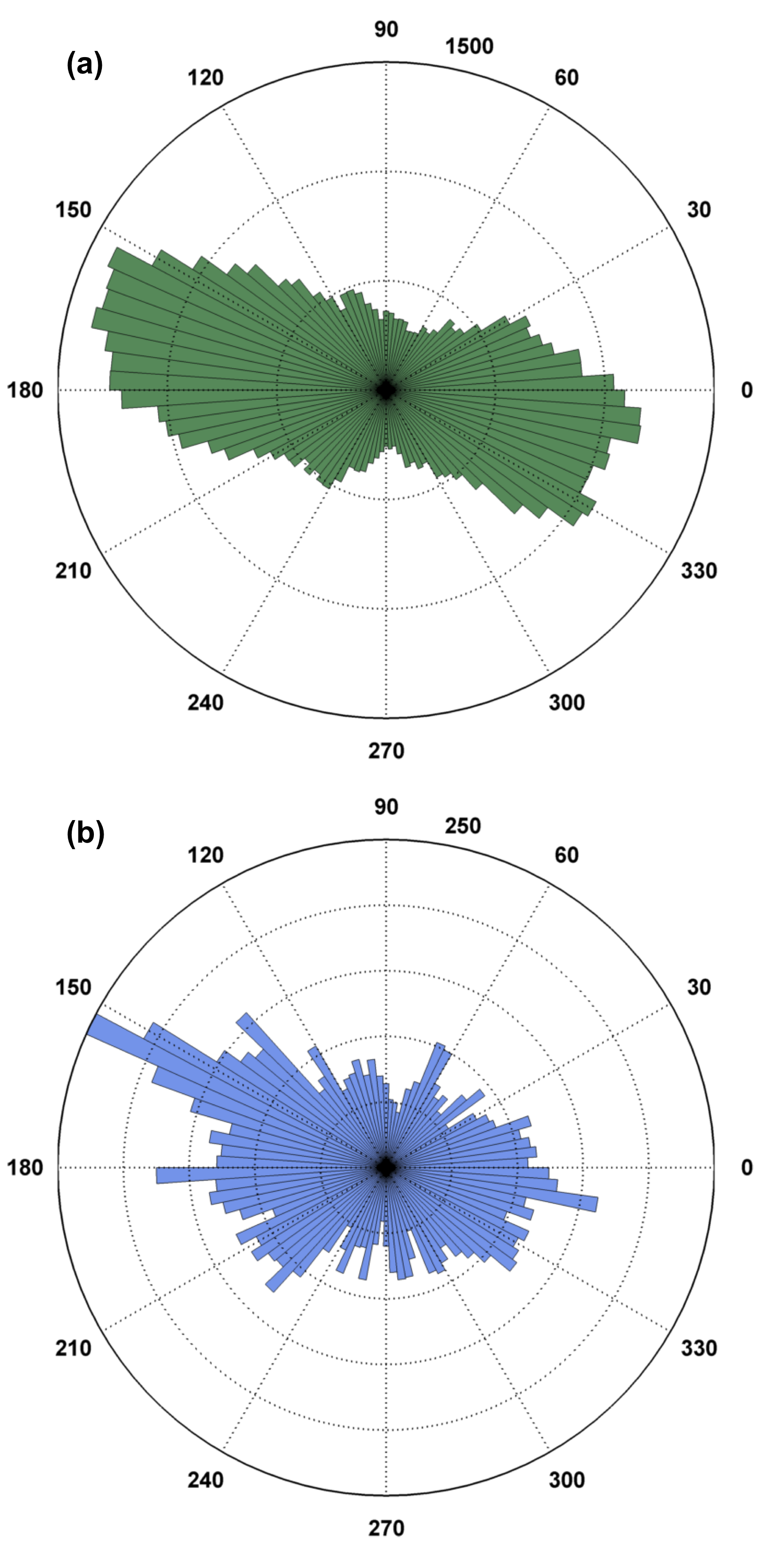

Supplement: S5 Fig — Two polar histograms showing all the recorded headings of the individually flying bats (a) and the paired flying bats (b) captured during the experiment. The line connecting the two recording cameras corresponds approximately to 180° and 0°. In both panels the measured headings, rather than being uniformly distributed, are biased towards mean angles. Computing the mean and standard deviations we obtain 349.4° and 169.4°, (±33.1°), for the individual flying bats and 348.0° and 168.0°, (±36.7°), for the paired flying bats. This bias might be caused by the bats trying to avoid the perimeter demarcating their foraging environment: one part along the shoreline where the cameras were mounted and the other part perpendicular to the latter and located just outside the recording region. (TIFF) [file pcbi.1004089.s006.tiff]

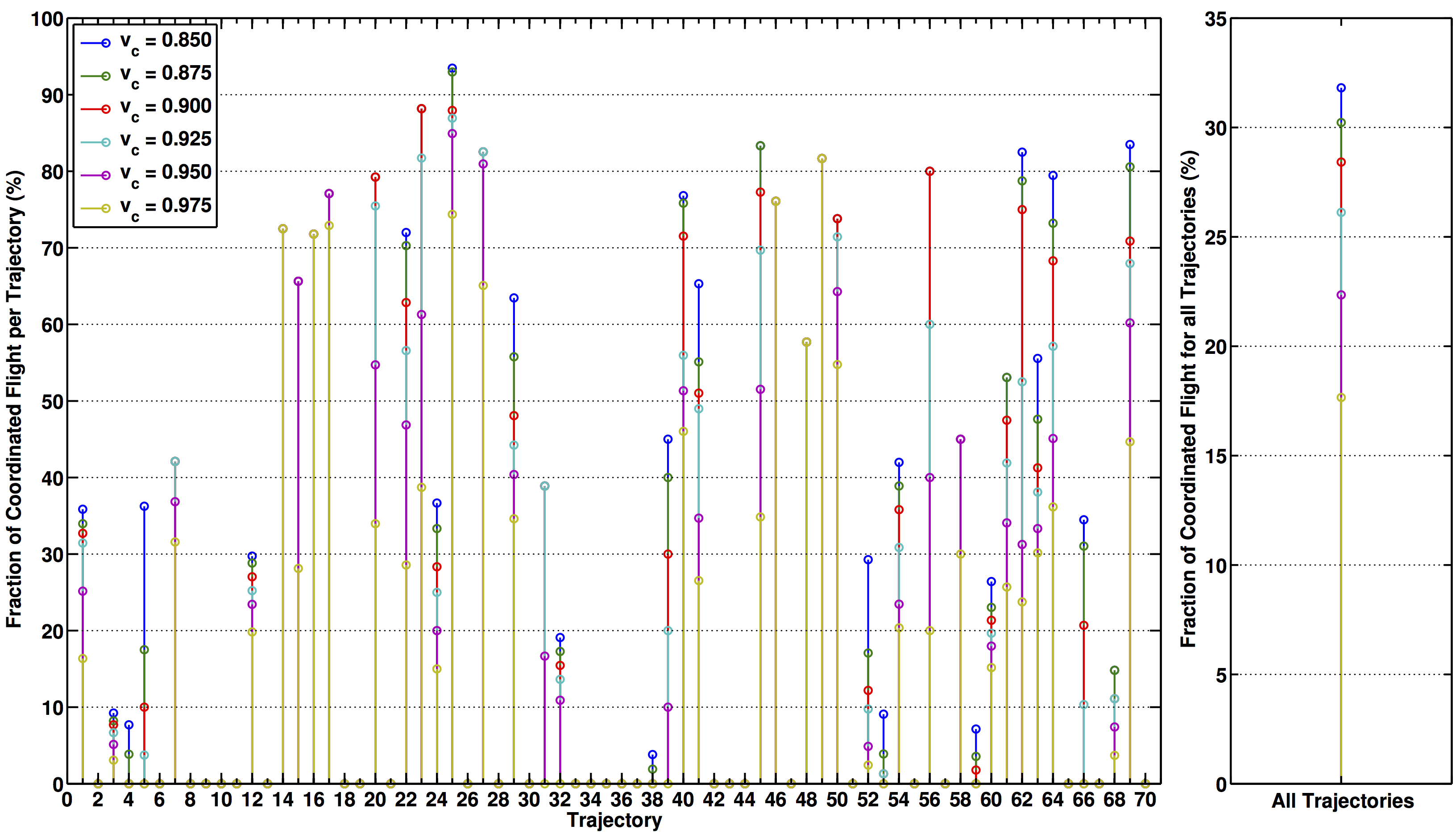

Supplement: S6 Fig — For each of the 70 trajectories that comprise the paired flight dataset, the percentage of that trajectory deemed interacting flight behaviour is shown for different correlation thresholds v c. The effect of changing v c is trajectory-dependent with almost no change in some cases, e.g. 14, and large variation in others, e.g. 56. An analysis of each of the 70 paired flights with thresholds smaller than the one used (0.85 ≤ v c < 0.95) reveals that only four trajectories (4, 38, 53, 59) brings about interacting behaviour that is otherwise missed. In all the other trajectories, a lower threshold simply increases the length of the interacting paths by converting unclassified segments to classified ones at the beginning or the end of a segment already deemed interacting. The right panel shows the total fraction of interacting flight behaviour for all the data for different thresholds v c. Trajectory 17, 20, 40 and 64 were depicted, respectively, in S2 Fig. panels (a) and (b), and S8 Fig. panels (a) (and also S1 Fig. panel(a)) and (b), whereas trajectory 7, 29 and 56, plotted respectively in S10 Fig. panels (a), (c) and (e), were deemed outliers and their entire dataset were catalogued as unclassified behaviour. (TIFF) [file pcbi.1004089.s007.tiff]

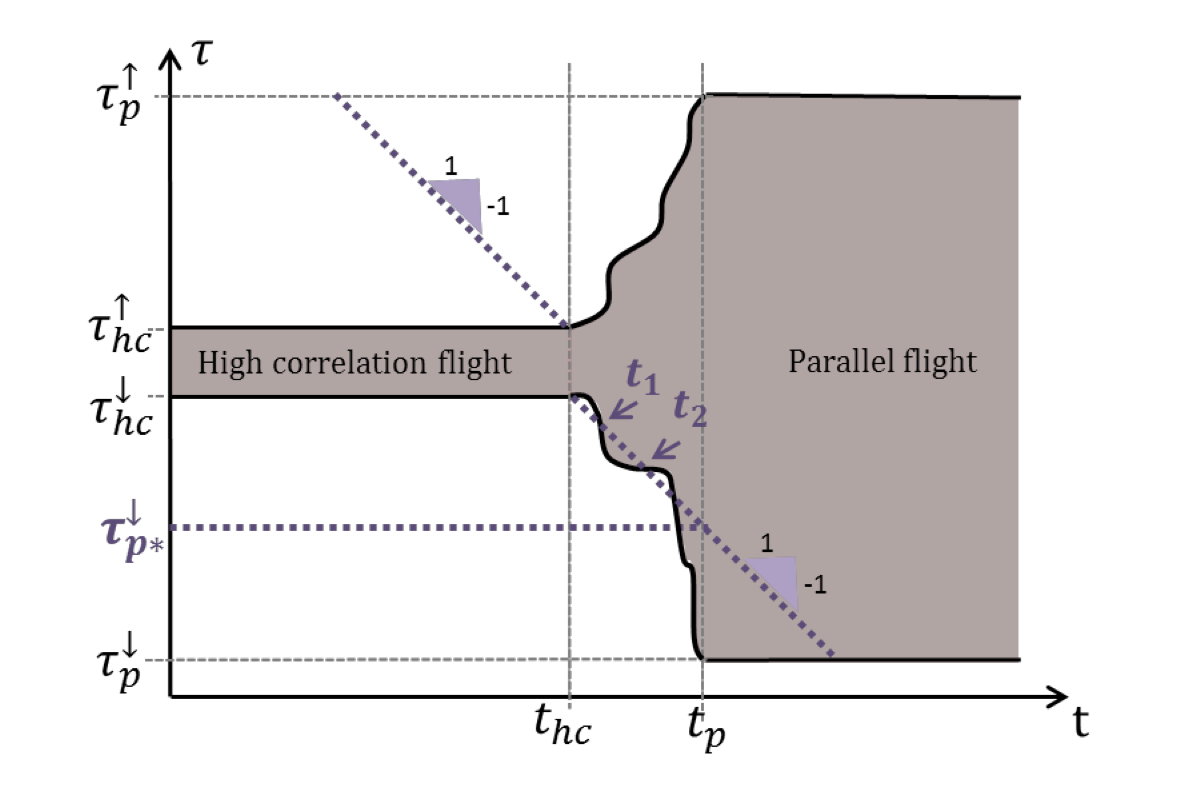

Supplement: S7 Fig — A schematic correlation map demonstrating the effect of trajectory curvature on delay extraction. Within the plot the correlations are distinguished by those below a selected threshold (white-space) and those above the threshold (shaded area), separated by the correlation threshold itself (black-line). Two distinct flight behaviours are drawn: high correlation flight where the bats are well aligned but turn frequently, and parallel flight where the bats fly in straight lines. Times and delays have been marked to demonstrate the effect of the time ordering condition (Δτ/Δt ≥ −1) on the uncertainty of delay extraction. (TIFF) [file pcbi.1004089.s008.tiff]

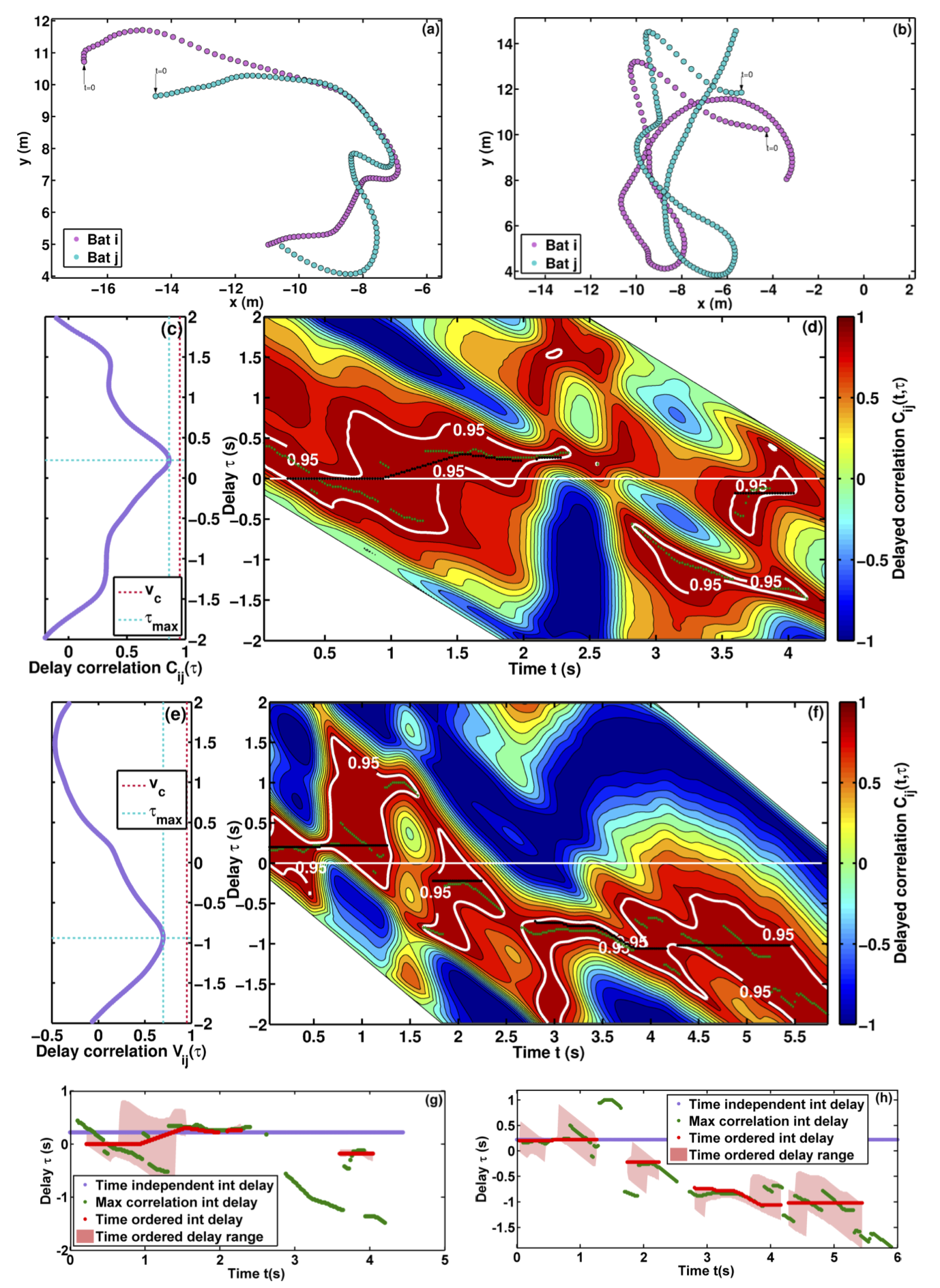

Supplement: S8 Fig — In panels (a) and (b) two sample trajectories are drawn with their corresponding TDDC functions in panels (d) and (f), respectively. The maximum of the TDDC function averaged over all times is shown in panel (c) and (e), respectively. In panel (d) and (f) we also show the delay path extracted from our time-ordered procedure (black line) and from selecting the so-called ‘maximal path’ [7], that is the maximum at each time of Equation (2). For panel (d) delay values (and uncertainties) for our time-ordered procedure between 1 s < t < 2 s are extracted by considering the TDDS plot (displayed in S1 Fig. panel (d)) when the interaction becomes a chase. A comparison of the delay values extracted with the different procedures from panel (a) and (b) is displayed, respectively, in panels (g) and (h) with shaded light red areas representing the uncertainty in the extracted delay from our combined analysis of the TDDC and TDDS function. (TIFF) [file pcbi.1004089.s009.tiff]

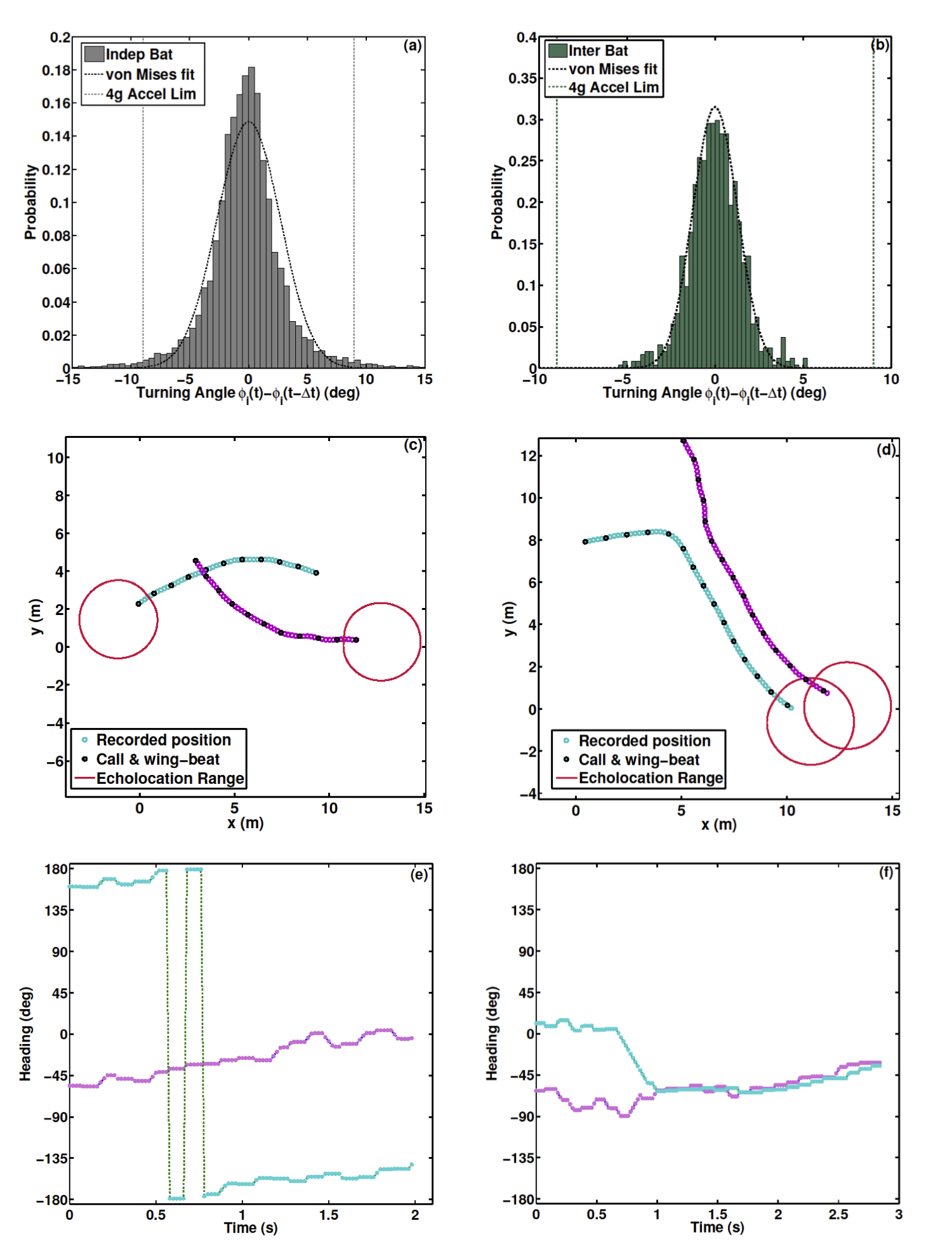

Supplement: S9 Fig — Panels (a) and (b) compare the distributions of turning angles for the observed unclassified and chase flights, respectively. A fit of the histograms with a von Mises distribution is also shown as well as the maximum lateral acceleration imposed on the model animals. Panels (c) and (d) show two examples of trajectories that emerge from the model in which the bats act independently and interactively. At the end of each trajectory we show the bats’ echolocation range obtained by selecting hearing threshold of 10 dB and an emission directionality with an asymmetric parameter A = 16 in Equation (11). Panels (e) and (f) show the bats’ headings corresponding to the coloured trajectories in (c) and (d), respectively. (TIFF) [file pcbi.1004089.s010.tiff]

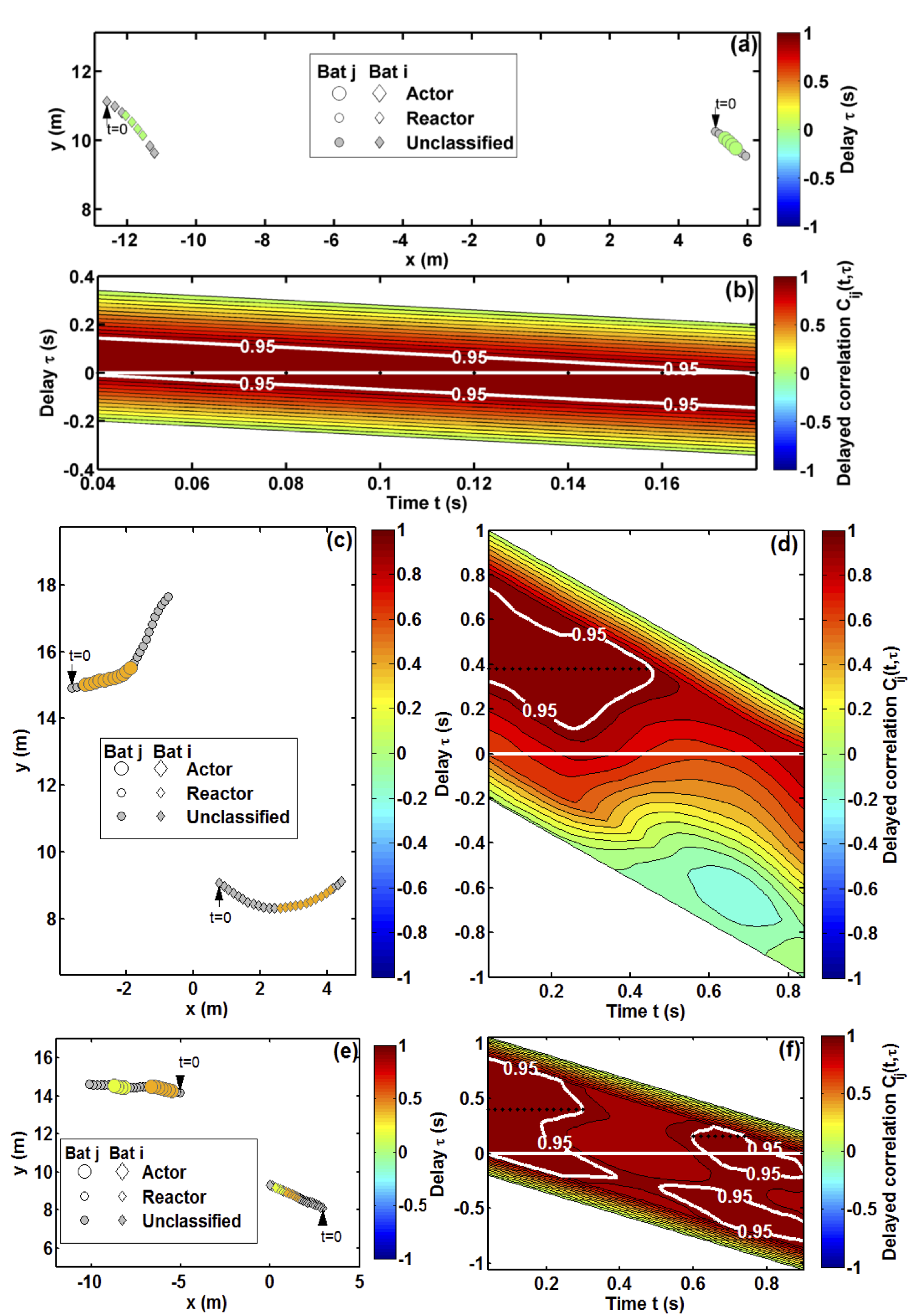

Supplement: S10 Fig — The paired panels (a)-(b), (c)-(d) and (e)-(f) show respectively the trajectory and correlation maps of bat pairs whose interactive behavioural identification was discarded because the animals’ separation distance was at the limit of their hearing range. (TIFF) [file pcbi.1004089.s011.tiff]
